# Impact of Artificial Sputum Medium Formulation on Pseudomonas aeruginosa Secondary Metabolite Production

**DOI:** 10.1128/JB.00250-21

**Published:** 2021-10-12

**Authors:** Rachel L. Neve, Brent D. Carrillo, Vanessa V. Phelan

**Affiliations:** a Department of Immunology and Microbiology, School of Medicine, University of Colorado, Anschutz Medical Campus, Aurora, Colorado, USA; b Department of Pharmaceutical Sciences, Skaggs School of Pharmacy and Pharmaceutical Sciences, University of Colorado, Anschutz Medical Campus, Aurora, Colorado, USA; Geisel School of Medicine at Dartmouth

**Keywords:** *Pseudomonas aeruginosa*, cystic fibrosis, metabolism, mucin, virulence factors

## Abstract

*In vitro* culture media are being developed to understand how host site-specific nutrient profiles influence microbial pathogenicity and ecology. To mimic the cystic fibrosis (CF) lung environment, a variety of artificial sputum media (ASM) have been created. However, the composition of these ASM vary in the concentration of key nutrients, including amino acids, lipids, DNA, and mucin. In this work, we used feature-based molecular networking (FBMN) to perform comparative metabolomics of Pseudomonas aeruginosa, the predominant opportunistic pathogen infecting the lungs of people with CF, cultured in nine different ASM. We found that the concentration of aromatic amino acids and iron from mucin added to the media contributes to differences in the production of P. aeruginosa virulence-associated secondary metabolites.

**IMPORTANCE** Different media formulations aiming to replicate *in vivo* infection environments contain different nutrients, which affects interpretation of experimental results. Inclusion of undefined components, such as commercial porcine gastric mucin (PGM), in an otherwise chemically defined medium can alter the nutrient content of the medium in unexpected ways and influence experimental outcomes.

## INTRODUCTION

Cystic fibrosis (CF) is a genetic disease characterized by the accumulation of thick and viscous bronchial mucus, which provides a nutrient-rich environment for the growth of opportunistic pathogens (1). Pseudomonas aeruginosa, the primary bacterial opportunistic pathogen infecting the lungs of people with CF, contributes to lung function decline by growing at high density within biofilms that are recalcitrant to the innate immune response and antibiotic therapy (2–4). While the nutritional environments of most sites of infection are poorly defined, the composition of CF pulmonary mucus is known to be comprised of amino acids, mucin, DNA, lipids, and micronutrients, including metals (5–9). To study the microbial physiology of P. aeruginosa and other pathogens *in vitro*, a variety of artificial sputum media (ASM) have been created that aim to replicate the nutrient availability of the CF lung (9–20). The various compositions of ASM were assembled from direct measurement of nutrients from clinical samples, personal observations, literature precedent, and economic cost of materials.

**FIG 1:**
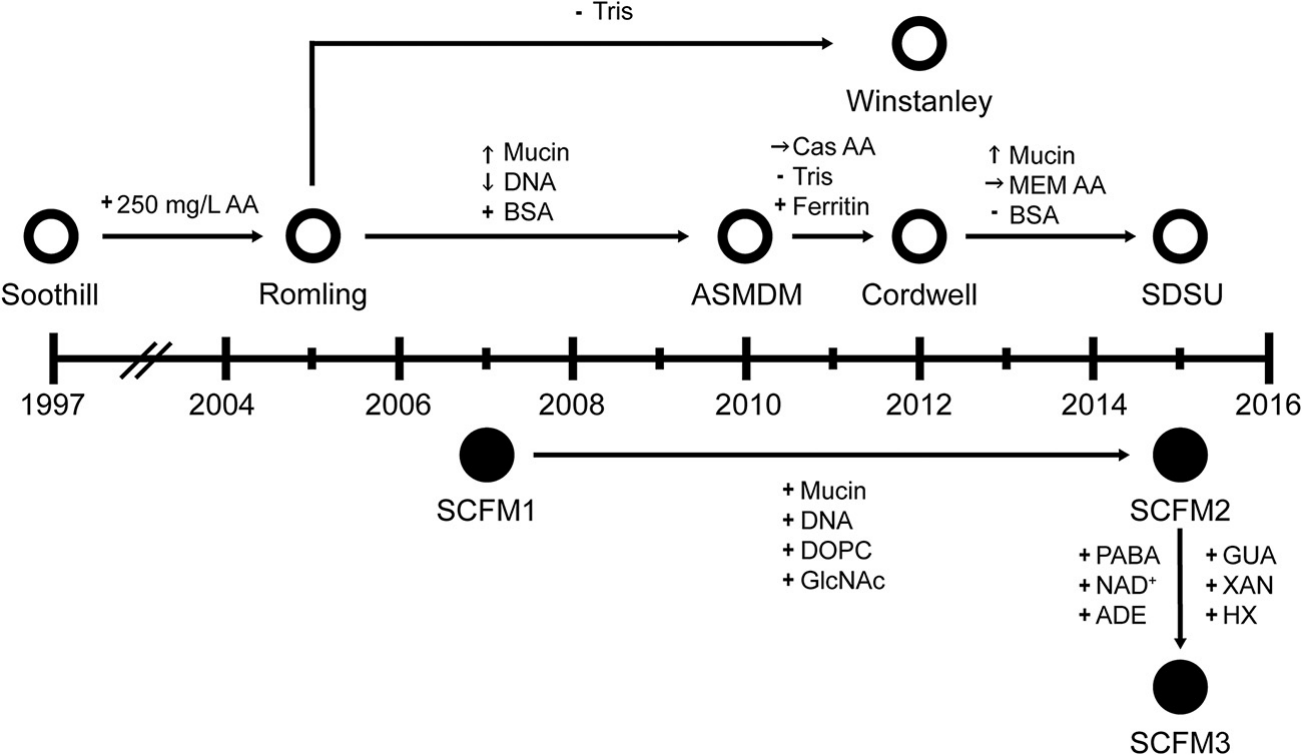
Publication timeline of ASM formulations. Each formulation is plotted at the year it was first published. Open circles indicate media derived from the Soothill ASM recipe. Closed circles represent the SCFM ASM lineage. Nutrient alteration between formulations are indicated by arrows. +, added; −, removed; ↑, increased; ↓, decreased; →, different source compared to the preceding formulations. AA, amino acids; BSA, bovine serum albumin; Cas AA, Casamino Acids; MEM AA, MEM amino acids; DOPC, 1,2-dioleoyl-*sn*-glycero-3-phosphocholine; GlcNAc, *N*-acetylglucosamine, PABA, 4-aminobenzoic acid; NAD^+^, nicotinamide adenine dinucleotide; ADE, adenine; GUA, guanine; XAN, xanthine; HX, hypoxanthine. Recipes for ASM formulations are in detailed in Data Set S1.

Nine ASM are regularly used to investigate microbial physiology, biofilm development, antibiotic susceptibility, and interspecies interactions in the context of CF (see Table S1 in the supplemental material) (9–17). These ASM can be separated into two lineages based upon the foundational formulations (Fig. 1; Table S2), Soothill-derived ASM and the synthetic CF medium (SCFM) series. Soothill ASM was established in 1997 from literature concentrations for key nutrients identified in CF sputum, including mucin, DNA, sodium, potassium, chloride, and lipids (10). Egg yolk emulsion was used as a source of lecithin, and the chelator diethylenetriamine pentaacetate (DTPA) was used to bind free iron in the medium, as most iron is bound to host proteins *in vivo*. Soothill ASM was modified by the addition of 250 mg/liter of each of the 20 canonical amino acids to generate Romling ASM, as amino acids are present in CF sputum (12). The ASM published by Kirchner et al. in 2012 (Winstanley) is compositionally identical to Romling except for its buffering capacity (15). To reflect recent measurements of nutrients from CF sputum, Romling ASM was altered to create ASMDM ASM by adding bovine serum albumin (BSA), increasing mucin concentration, and reducing DNA levels (13). Derived from ASMDM ASM, Cordwell ASM has reduced buffering capacity, Casamino Acids (Cas AA) in place of individual amino acids, and ferritin instead of DTPA to replicate CF sputum iron availability (14). Cordwell ASM was modified via substitution of minimal essential medium (MEM) amino acids (MEM AA) for Cas AA, removal of BSA, and an increase in mucin concentration to make San Diego State University (SDSU) ASM (16).

Chemically defined SCFM1 was developed in 2007 from the average concentrations of ions, free amino acids, glucose, and lactate measured from CF sputum samples (9). SCFM1 was supplemented with DNA, mucin, *N*-acetylglucosamine (GlcNAc), and dioleoylphosphatidylcholine (DOPC) at concentrations similar to those measured from CF sputum to create SCFM2 (17). Interrogation of P. aeruginosa strain-specific genetic requirements for *in vitro* growth using sputum as a medium identified a set of genes located in the anabolic pathways for the biosynthesis of thiamine, nicotinamide adenine dinucleotide (NAD), purines, folate, branched-chain amino acids, and tryptophan. These results suggested that these metabolites are more available in CF sputum than SCFM2. Therefore, SCFM2 was further modified by the addition of *p*-aminobenzoic acid, NAD^+^, adenine, guanine, xanthine, and hypoxanthine to generate SCFM3 (17).

Although each of the nine ASM formulations is distinct in composition, experimental conclusions from different ASM claim to provide depictions of pathogen growth and physiology that are reflective of observations made from patient samples (12–14, 21–30). However, disparate nutrient concentration and bioavailability between ASM formulations likely affect microbial physiology and production of virulence factors. Therefore, we hypothesized that the compositional variations between ASM would be sufficient to promote differential production of P. aeruginosa secondary metabolites. These virulence-associated metabolites play important roles in mediating the interactions of P. aeruginosa with host cells and other microbes as well as acquisition of nutrients from the environment (31).

Herein, we describe the application of comparative metabolomics to P. aeruginosa PAO1 stationary biofilm cultures grown in nine ASM formulations. (9–17). PAO1 is a commonly used laboratory strain whose secondary metabolite production has been well documented (32, 33). We show that differential production of P. aeruginosa secondary metabolites between ASM formulations is driven in part due to variations in aromatic amino acid concentration as well as high concentrations of iron added to the media from the inclusion of commercial porcine gastric mucin (PGM). Importantly, due to their roles in virulence-associated phenotypes, altered secondary metabolite production by P. aeruginosa due to compositional differences likely influences the outcome of microbe-microbe interactions, virulence assays, and comparative assessment of experiments performed in different ASM. Additionally, the influence of crude complex nutrients, like mucin, on experimental outcomes may not be due to the nutrient itself but, rather, other constituents of the mixture.

## RESULTS

### ASM formulation affects P. aeruginosa PAO1 growth.

**FIG 2:**
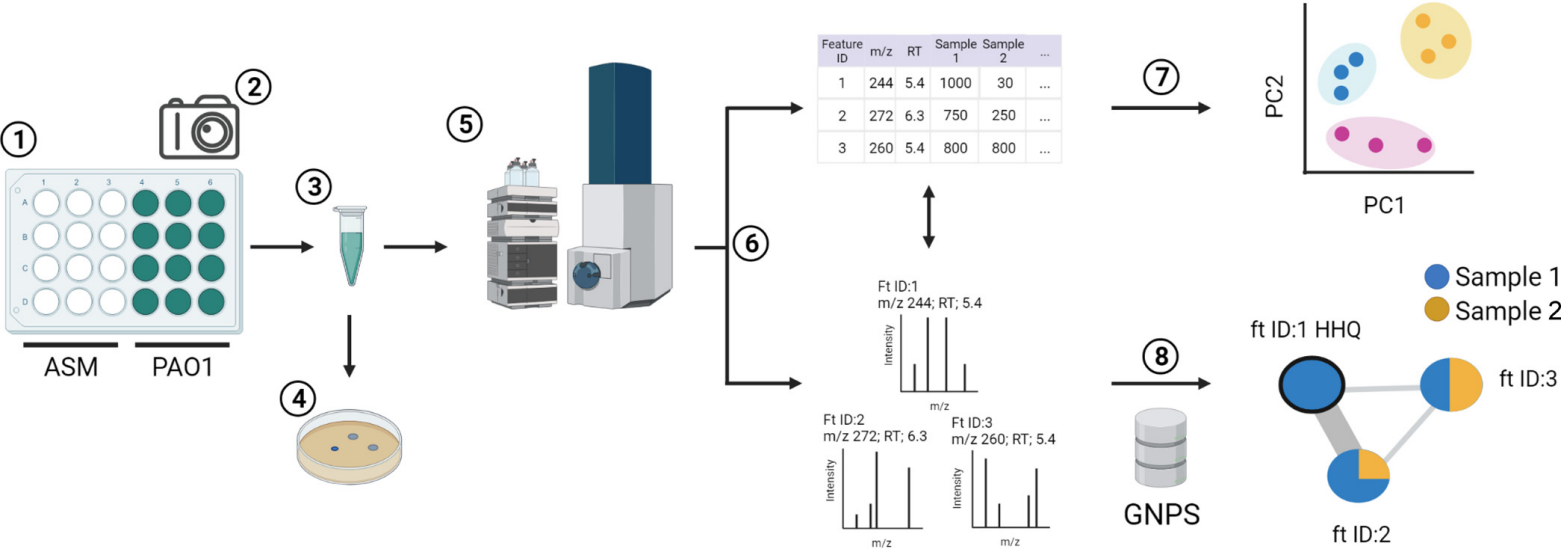
Experimental workflow. (1) P. aeruginosa PAO1 growth and secondary metabolite production in artificial sputum media (ASM) was evaluated in 24-well plates (12 control wells and 12 growth wells per medium). The media were inoculated in the following three batches: batch 1, LB, Soothill, SDSU, and Cordwell ASM; batch 2, LB, Romling, Winstanley, ASMDM, and SCFM1 ASM; and batch 3, LB, SCFM2, and SCFM3 ASM. All plates were covered and incubated statically at 37°C for 72 h. (2) Growth and control wells were photographed. (3) Samples from each batch were mechanically disrupted and aliquoted. (4) A subset of aliquots (*n* = 3) was used to measure growth (CFU per milliliter) and culture pH. (5) Aliquots of control and growth samples (*n* = 12) were chemically extracted for metabolomics analysis and subjected to liquid chromatography-tandem mass spectrometry (LC-MS/MS). (6) Metabolomics data from growth samples were processed for feature-based molecular networking (FBMN) using MZmine 2.0. This data processing yielded two complementary summary files, a feature quantification table and MS/MS spectral summary. The feature quantification table provided molecular ion abundance across all samples. The MS/MS spectral summary provided a representative MS/MS spectrum for each molecular ion in the feature quantification table and was used to determine compound identity using molecular networking. (7) MetaboAnalyst v5.0 was used to analyze the feature quantification table using statistical methods. (8) The MS/MS spectral summary was analyzed via the Global Natural Products Social Molecular Networking (GNPS) online data analysis platform using the FBMN workflow to organize structurally related features into molecular families using MS/MS spectral similarity as a proxy for structural similarity. Spectral similarity was also used to annotate features as metabolites based on matches to the GNPS spectral libraries. Abundance data from the feature quantification table was overlaid onto the molecular network as pie charts to visually compare the relative amount of each feature present in samples from each ASM formulation.

**FIG 3:**
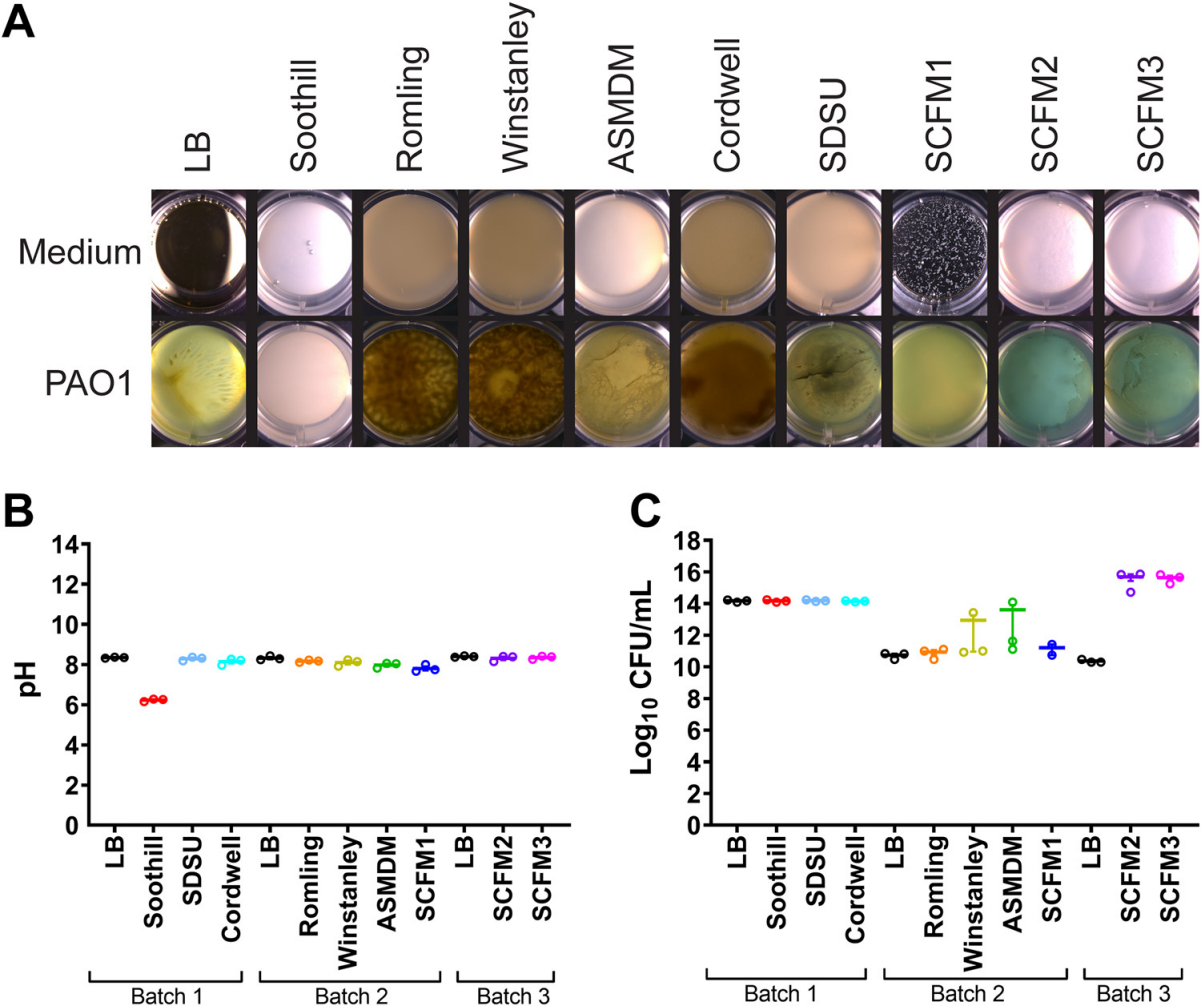
P. aeruginosa PAO1 growth in ASM. (A) Representative phenotypes of PAO1 grown statically in 2 ml of each ASM formulation (*n* = 12) in 24-well plates for 72 h at 37°C. Photographs of replicate control and growth wells are in Fig. S1 in the supplemental material. (B) pH measurement of representative replicates (*n* = 3) after mechanical disruption of growth in ASM. (C) Representative replicates of PAO1 growth (*n* = 2 or 3) in ASM.

To assess the effect of ASM composition on growth, strain PAO1 was cultured in ASM (Fig. 2). Samples were grown and evaluated in the following three batches, with one plate per medium: batch 1, which includes LB, Soothill, SDSU, and Cordwell ASM; batch 2, which includes LB, Romling, Winstanley, ASMDM, and SCFM1 ASM; and batch 3, which includes LB, SCFM2, and SCFM3 ASM. Gross morphology of the biofilms was visualized using a stereo microscope. As expected, the cultures varied in structure and color depending upon the growth medium (Fig. 3A; see Fig. S1 and Table S3 in the supplemental material). Among ASM formulations, PAO1 formed apparent biofilm structures in all but Soothill ASM. The translucent but turbid cultures in Soothill ASM indicate that the lack of exogenous amino acids affects both secondary metabolite production and biofilm formation. Due to their nearly identical compositions, Romling and Winstanley ASM cultures grew as brown macrostructures suspended in solution. The nutrient alterations from Romling to ASMDM ASM strongly influenced the P. aeruginosa phenotype, resulting in a tan-green macrostructure at the air-liquid interface, with large open pores within the structure giving way to turbid growth toward the bottom of the well. Surprisingly, the composition of Cordwell ASM induced a phenotype similar to those observed in Romling and Winstanley ASM; PAO1 grew as a dense brown biofilm without clear structural features. Biofilms in SDSU ASM grew as a light-green structure with tendrils near the center of the well that reached the surface of the medium. For SCFM-series ASM, growth in SCFM1 was turbid and bright green, which was noticeably distinct from biofilms in SCFM2 and SCMF3, which were comparable in structure with film-like growth at the air-liquid interface, aggregates on the bottom of the well, and a marked blue color.

Importantly, gradients of pH have been shown to dictate microbial niche partitioning in *in vitro* studies (34). The phenotypic differences between ASM cultures were not driven by variations in pH. As these cultures were incubated statically, the lack of flow of nutrients in our system may have led to a buildup of metabolites that increase the pH (Fig. 3B). The alkalinity of samples after PAO1 growth and sample disruption suggests the nutritional profile of ASM or the culture conditions may not fully replicate the *in vivo* environment. Notably, only Soothill ASM cultures became acidic, which suggests that metabolites produced from amino acids may contribute to changes in pH.

PAO1 growth in ASM was enumerated as CFU per milliliter (Fig. 3C). While growth was robust, quantification was impacted by batch effects. In batch 1 (LB, Soothill, SDSU, and Cordwell ASM), P. aeruginosa grew to ∼10^14^ CFU/ml. However, growth in batch 2 (LB, Romling, Winstanley, and ASMDM ASM) was measured at ∼10^10^ CFU/ml. As biofilm disruption was affected by culture viscosity and aggregation, it is impossible to distinguish whether the difference in growth between batches is due to ASM composition or processing of samples.

### Identification of secondary metabolites produced in ASM.

Although several P. aeruginosa secondary metabolites have authentic commercial standards, most must be identified using other methods, such as spectral matching to metabolite databases. Therefore, to quantify and identify the secondary metabolites produced by PAO1 in ASM, we performed feature-based molecular networking (FBMN) on untargeted liquid chromatography-tandem mass spectrometry (LC-MS/MS) metabolomics data (Fig. 2; Table S4). (35, 36). FBMN combines the quantitative assessment of feature finding from traditional metabolomics analysis with MS/MS molecular networking, which organizes metabolites into structurally related molecular families. Feature finding is a data reduction step in LC-MS analysis which creates a quantitative representation (feature table) of all molecular ions, or features, within a metabolomics data set. Each feature is defined by its precursor mass, retention time, and corresponding abundance. In data processing for FBMN, an additional step is applied to the LC-MS/MS data to provide an MS/MS spectral summary in which each feature is associated with an MS/MS spectrum. The MS/MS spectra in the spectral summary are then networked using the Global Natural Products Social Molecular Networking (GNPS) web-based platform (36). Based upon the assumption that molecules of similar structure will have similar MS/MS fragmentation patterns, molecular networking (MN) organizes the MS/MS data into molecular families based upon their spectral similarity. Within the molecular network, nodes (or clusters) represent a feature, and the thickness of the connecting lines (or edges) indicates the level of MS/MS spectral similarity between the features. The MS/MS spectra in the data set are simultaneously scored for their relatedness to the GNPS spectral libraries to provide initial identification of metabolites. The abundance of each feature in each sample group (e.g., individual ASM formulations) is overlaid onto the molecular network as a pie chart, wherein the different colors represent the different samples groups, and the size of a wedge indicates the relative abundance of that feature.

**FIG 4:**
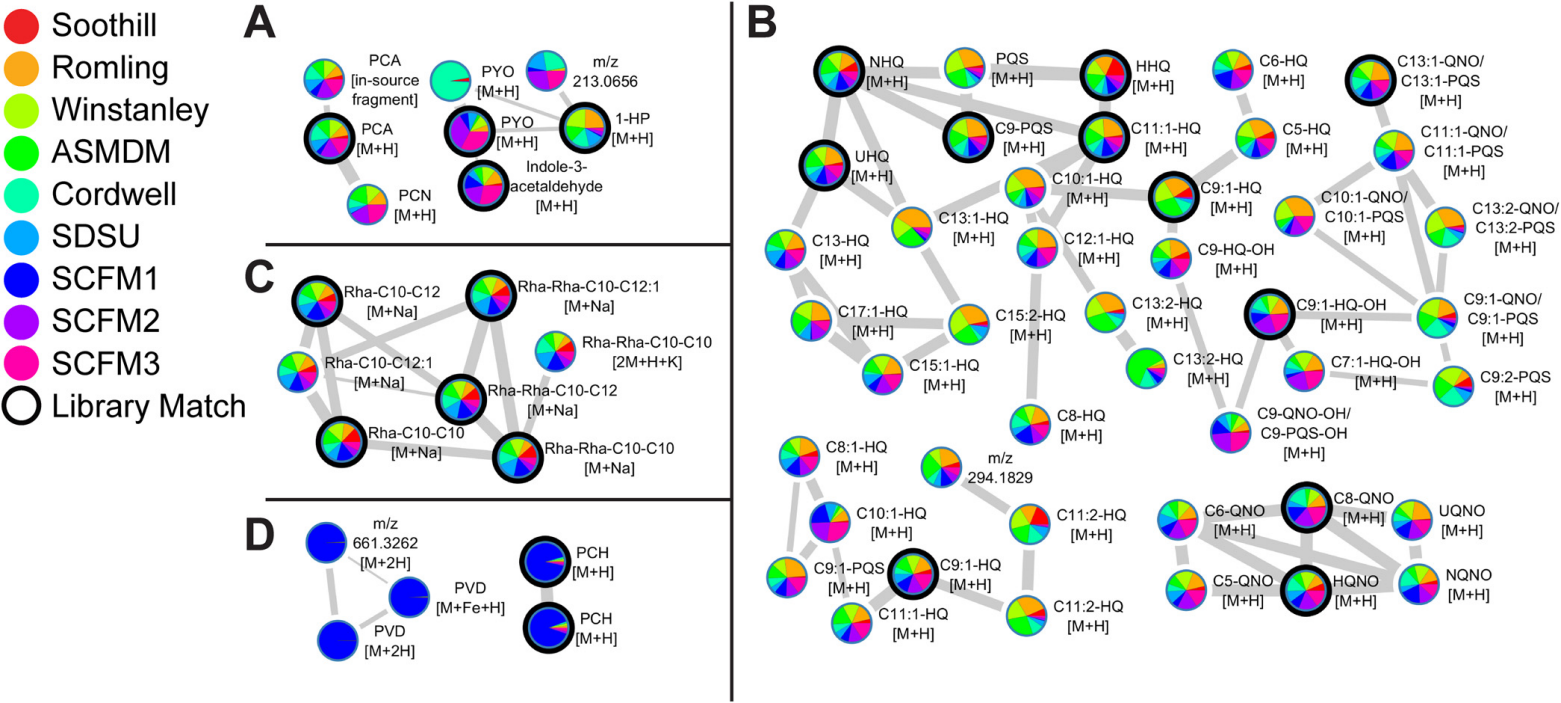
Molecular annotation and relative production of PAO1 secondary metabolites using FBMN. Molecular families of P. aeruginosa secondary metabolites (as determined by MS/MS) identified in the feature-based molecular network are shown. Data includes extracts from PAO1 cultures in all ASM formulations (*n* = 12 biological replicates per formulation). Nodes represent distinct features (precursor mass and LC retention time). The width of the edges represent the similarity of the MS/MS fragmentation of the connected nodes, while the node color represents the relative feature abundance between ASM formulations. Black circles indicate a match between the data and the GNPS spectral libraries. Nodes annotated as compounds are labeled with an abbreviated compound name. Nodes that could not be assigned structures are labeled with the precursor mass of the feature. The adduct ions detected are indicated for all nodes. (A) Phenazine molecular family. 1-HP, 1-hydroxyphenazine; PYO, pyocyanin; PCN, phenazine-1-carboxamide; PCA, phenazine-1-carboxylic acid. (B) Quinolone molecular family. HHQ, 2-heptyl-4-quinolone; NHQ, 2-nonyl-4-quinolone; UHQ, 2-undecyl-4-quinolone; PQS, 2-heptyl-3-hydroxy-4-quinolone; HQNO, 2-heptyl-4-hydroxyquinoline *N*-oxide; NQNO, 2-nonyl-4-hydroxyquinoline *N*-oxide; UQNO, 2-undecyl-4-hydroxyquinoline *N*-oxide. All other nodes in the molecular family are annotated by alkyl chain length (e.g., C_5_, alkyl chain containing 5 carbons) and unsaturation (e.g., C_9:1_, alkyl chain containing 9 carbons and 1 double bond), followed by structural subclass assignment (-HQ, -PQS, and -QNO) to denote structural relatedness to HHQ, PQS, and HQNO, respectively. The structural subclass of nodes labeled with QNO/PQS could not be distinguished based upon MS/MS fragmentation pattern. Annotations with -OH designation indicate hydroxylation at unknown position within the molecule. (C) Rhamnolipid molecular family. Nodes corresponding to rhamnolipids are annotated by rhamnose units (Rha) followed by fatty acid chain lengths (e.g., Rha-C_10_-C_10_ is a rhamnolipid containing one rhamnose unit and two fatty acids containing 10 carbons). (D) Pyoverdine (PVD; left) and pyochelin (PCH; right) molecular families.

In total, 492 features were quantified and visualized in the molecular network (Fig. S2). Of those features, 80 (16%) had spectral matches to the GNPS libraries with 30 (6%) to phenazine, quinolone, rhamnolipid, and pyochelin reference spectra. Of the five phenazines produced by PAO1, 1-hydroxyphenazine (1-HP), pyocyanin (PYO), phenazine-1-carboxamide (PCN), and phenazine-1-carboxylic acid (PCA) were quantified (Fig. 4A) (37). From the pie chart representation of relative abundance, PYO levels reflect the blue color of SCFM2 and SCFM3, while 1-HP levels were highest in Romling, Winstanley, and ASMDM ASM.

Of over 50 quinolones reported in literature, 43 were quantified from ASM cultures, including functionally characterized 2-heptyl-4-quinolone (HHQ), 2-heptyl-3-hydroxy-4-quinolone (Pseudomonas quinolone signal; PQS), and 2-heptyl-4-hydroxyquinoline *N*-oxide (HQNO) (Fig. 4B) (38, 39). As most of the structural variation of the quinolones was due to differences in alkyl chain length, the quinolones quantified were categorized into structural subclasses based upon their MS/MS spectral similarity to HHQ, PQS, and HQNO (38, 40). Twenty-three quinolones were categorized as HHQ-type, six were grouped as HQNO-type, and four were assigned as PQS-type quinolones. Twelve nodes could not be assigned to a specific structural subclass from the experimental data. The relative quantification displayed on the nodes demonstrated that PAO1 quinolone production was heterogeneous among ASM and did not align with substructure categories.

Structurally, P. aeruginosa rhamnolipids consist of 1 to 2 rhamnose units bound to 3‐(3‐hydroxyalkanoyloxy) alkanoic acids (HAAs), yielding mono- and di-rhamnolipids, respectively (41). Six rhamnolipids were identified, including three mono-rhamnolipids and three di-rhamnolipids with fatty acid HAAs of C_10_-C_10_, C_10_-C_12:1_, and C_10_-C_12_ (Fig. 4C). Unlike phenazine and quinolone levels, all rhamnolipids showed similar production patterns between ASM cultures.

Nodes in the molecular network were identified as pyochelin by a spectral match to the GNPS libraries (Fig. 4D). As illustrated in the pie chart visualization, pyochelin was primarily produced by PAO1 in SCFM1. Other nodes with similar coloration within the molecular network were prioritized for identification, as the production pattern suggested that these nodes corresponded to features related to pyochelin or under similar regulation. Manual assessment of the precursor masses, MS/MS spectra, and retention times led to the identification of pyoverdine E (succinamide isoform) and Fe^3+^-bound pyoverdine E (40). The identification of *m/z* 661.3262 could not be determined from the experimental data. Further analysis of the FBMN also identified ferribactin (succinamide isoform), the nonribosomal peptide cytoplasmic precursor to pyoverdines (Table S4) (42). Although pyoverdine biosynthesis was reported to be upregulated in P. aeruginosa SCFM1 cultures, the production of the pyoverdine molecular family by PAO1 is below the limit of detection when cultured in most media for the LC-MS/MS method used in this study (9).

### PAO1 metabolome changes in response to ASM formulation.

To determine which molecular features differentiated the metabolome of PAO1 in different ASM, the feature table was analyzed using principal-component analysis. From the initial results (Fig. S3), the clustering of samples in the scores plot was due to batch effects, primarily driven by the abundance of benzethonium in batch 1 samples. Benzethonium is an ingredient of bactericidal surface disinfectants. As this compound was introduced during sample processing, it was removed from the data set, and the data were reanalyzed.

**FIG 5:**
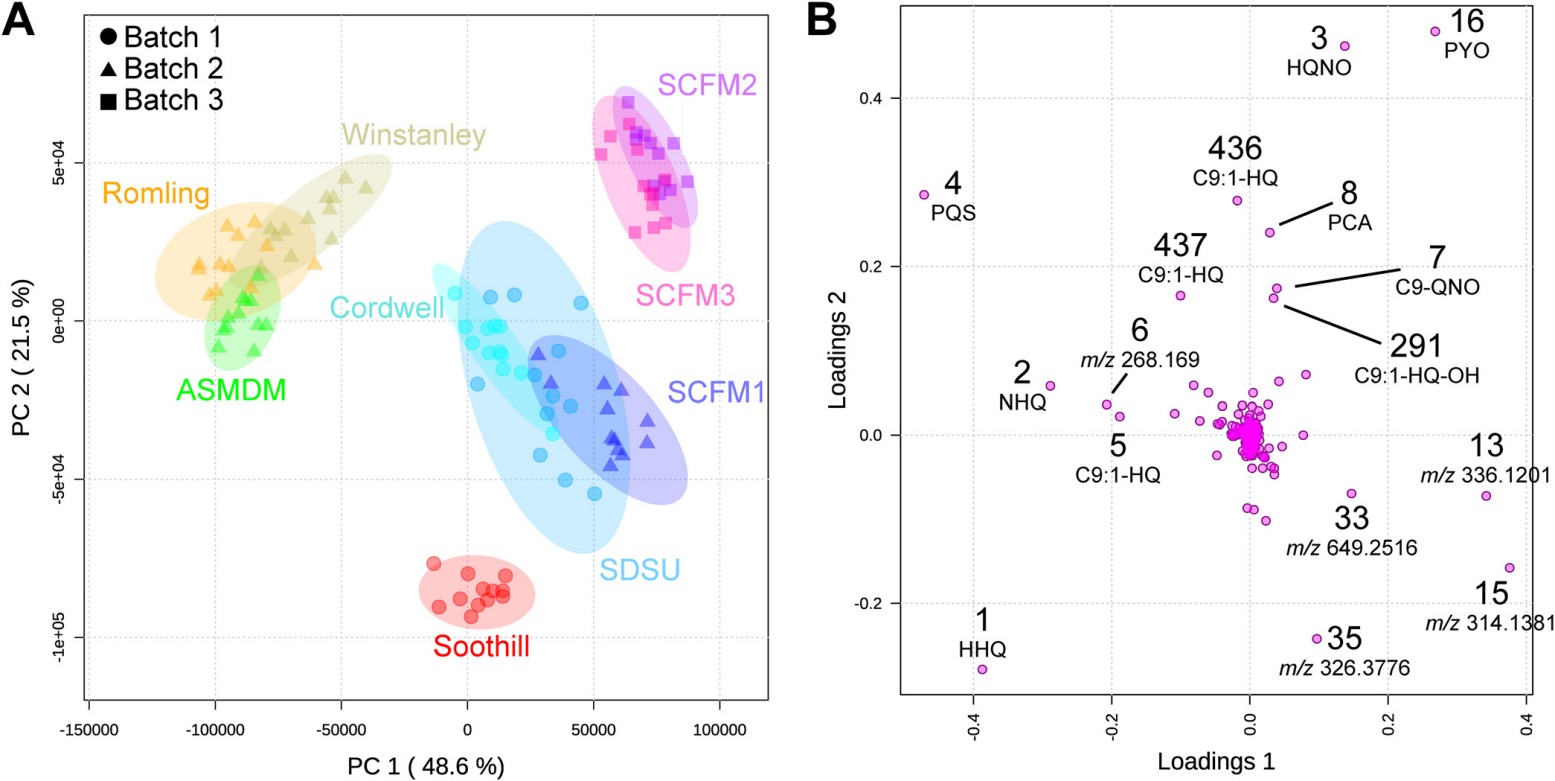
Principal-component analysis and its loadings plots of untargeted metabolomics data of PAO1 grown in ASM formulations. (A) Principal-component analysis scores plot of PAO1 samples, colored by medium and drawn with 95% confidence ellipses. The three processing batches are indicated by shapes. Data points representing the 12 replicate samples per medium were closely clustered and distinct clusters were observed for metabolite profiles from cultures in (i) Romling, Winstanley, and ASMDM ASM; (ii) SCFM2 and SCFM3; (iii) Cordwell, SDSU, and SCFM1 ASM; and (iv) Soothill ASM. (B) Loadings plot from untargeted LC-MS/MS-based principal-component analysis of PAO1 in ASM formulations. Features are labeled by their identification number from the feature quantification table and were identified using the FBMN results (Fig. 4). Features of unknown structure are labeled with their precursor mass. Features along the *x* axis (loadings 1) were responsible for the separation observed along the horizontal axis (PC1) of panel A. Features along the *y* axis (loadings 2) were responsible for the separation observed along the vertical axis (PC2) of panel A.

A principal-component model was created with five principal components. Extracts of replicate cultures grown in the same ASM clustered together within the two-dimensional space created by principal component 1 (PC1) and principal component 2 (PC2) of the scores plot, which explained 48.6% and 21.5% of the variation between samples, respectively (Fig. 5A). Four sample groups emerged from the principal-component analysis. Soothill ASM samples clustered within their own group. Samples from compositionally related media clustered together, with Romling, Winstanley, and ASMDM ASM samples in one group and samples from SCFM2 and SCFM3 in another. The fourth group consisted of samples from Cordwell, SDSU, and SCFM1 ASM. However, this grouping was influenced by the two-dimensional projection of the data as these media separate in principal component 3 (PC3). This result indicated that the metabolite profiles of replicates of PAO1 cultures in individual ASM are highly similar, and, unsurprisingly, nutrient composition of the ASM is likely the primary driver of differences in PAO1 secondary metabolite production.

**FIG 6:**
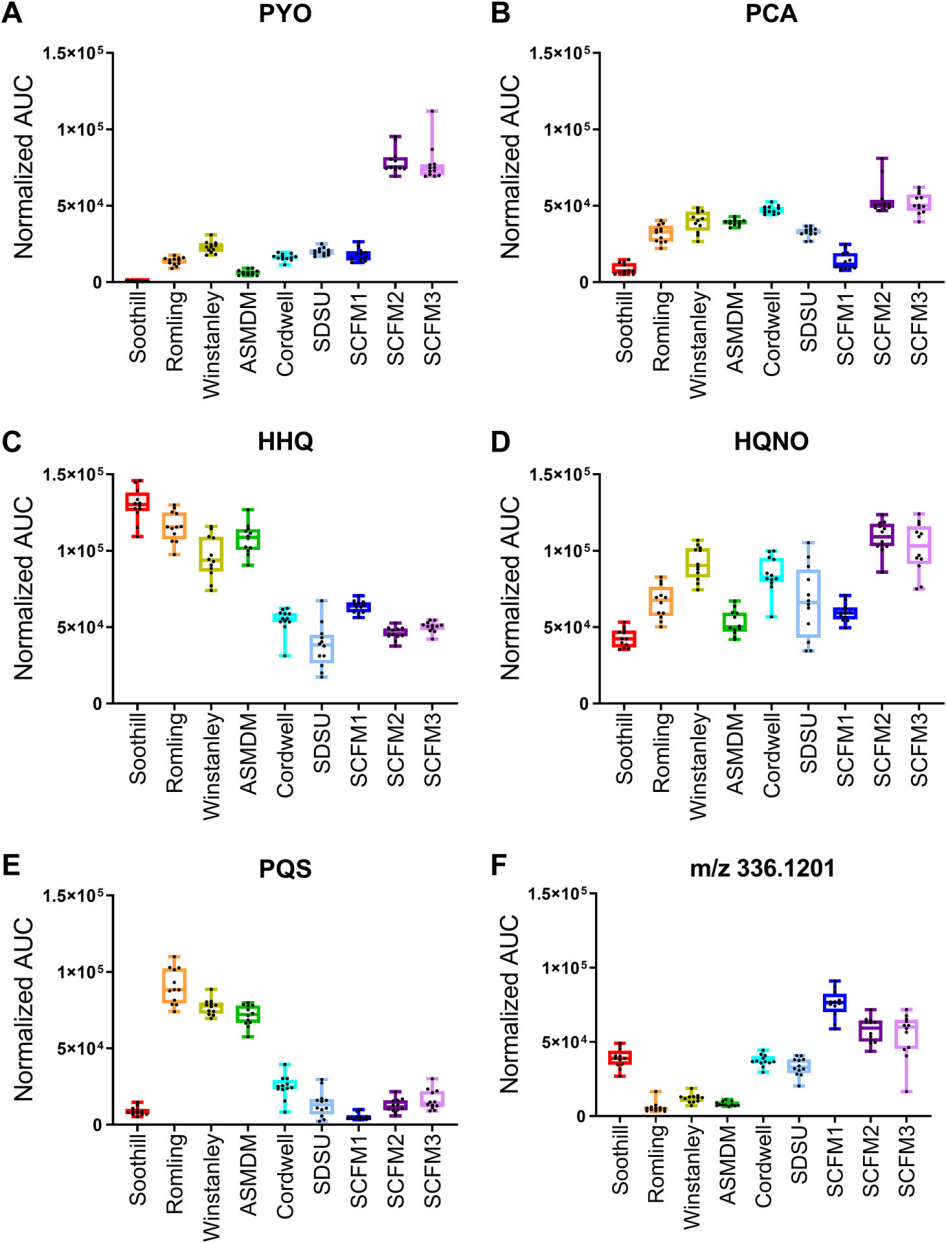
Differential levels of features in ASM formulations identified in the principal-component analysis. Total normalized area under the curve (AUC) for metabolites identified from the principal-component analysis in samples from PAO1 grown in each ASM. Box plots represent the25 to 75th percentiles, with a line at the median. Error bars indicate the minimum to maximum. Individual sample values shown (*n* = 12 biological replicates per ASM formulation). (A) Pyocyanin (PYO). (B) Phenazine-1-carboxylic acid (PCA). (C) 2-heptyl-4-quinolone (HHQ). (D) 2-heptyl-4-hydroxyquinoline *N*-oxide (HQNO). (E) Pseudomonas quinolone signal (PQS). (F) A feature of unknown structure with a precursor mass of 336.1201.

Principal-component loadings estimate the extent to which each metabolite contributes to each of the principal components, with magnitude in the two-dimensional space signifying the contribution of a specific metabolite to sample clustering in the scores plot (Fig. 5B). The separation of sample groups in the scores plot is due to several highly abundant metabolites, including PYO, PCA, HHQ, HQNO, PQS, and a set of unknown metabolites, including feature 13 (*m/z* 336.1201; C_18_H_19_NO_4_ [M+Na]). PYO contributed positively to the separation of both PC1 and PC2, suggesting that its abundance influenced the clustering of SCFM2 and SCFM3 samples from the other ASM (Fig. 6A). PCA also contributed positively to PC2 but to a lesser extent than PYO. Lower production of PCA by PAO1 in Soothill and SCFM1 promotes separation of these samples along PC2 (Fig. 6B). Feature 13 and other related unknown metabolites were detected in smaller amounts in Romling, Winstanley, and ASMDM samples (Fig. 6C) contributing to separation along PC1.

While multiple quinolones promoted grouping of ASM samples in the scores plot, HHQ, HQNO, and PQS abundance had the largest effect. HHQ abundance was highest in Soothill, Romling, Winstanley, and ASMDM samples, which contributed to the separation of these ASM along both PC1 and PC2 (Fig. 6D). The amplitude of HQNO in the loading plot suggested that it contributed positively to sample separation along PC2, with a smaller effect along PC1. HQNO abundance was higher in Winstanley, Cordwell, SCFM2, and SCFM3 ASM cultures than in Soothill, Romling, ASMDM, and SCFM1 ASM cultures (Fig. 6E). The lack of tight clustering of SDSU samples in the scores plot is partially explained by the variability of HQNO measured. PQS contributed negatively to PC1 and positively to PC2, aligning with the position of Romling, Winstanley, and ASMDM samples in the left upper quadrant of the scores plot. Confirming the impact of PQS on the sample clustering within scores plot, PQS abundance was higher in Romling, Winstanley, and ASMDM cultures (Fig. 6F).

### ASM formulation influences PAO1 secondary metabolite family production.

**FIG 7:**
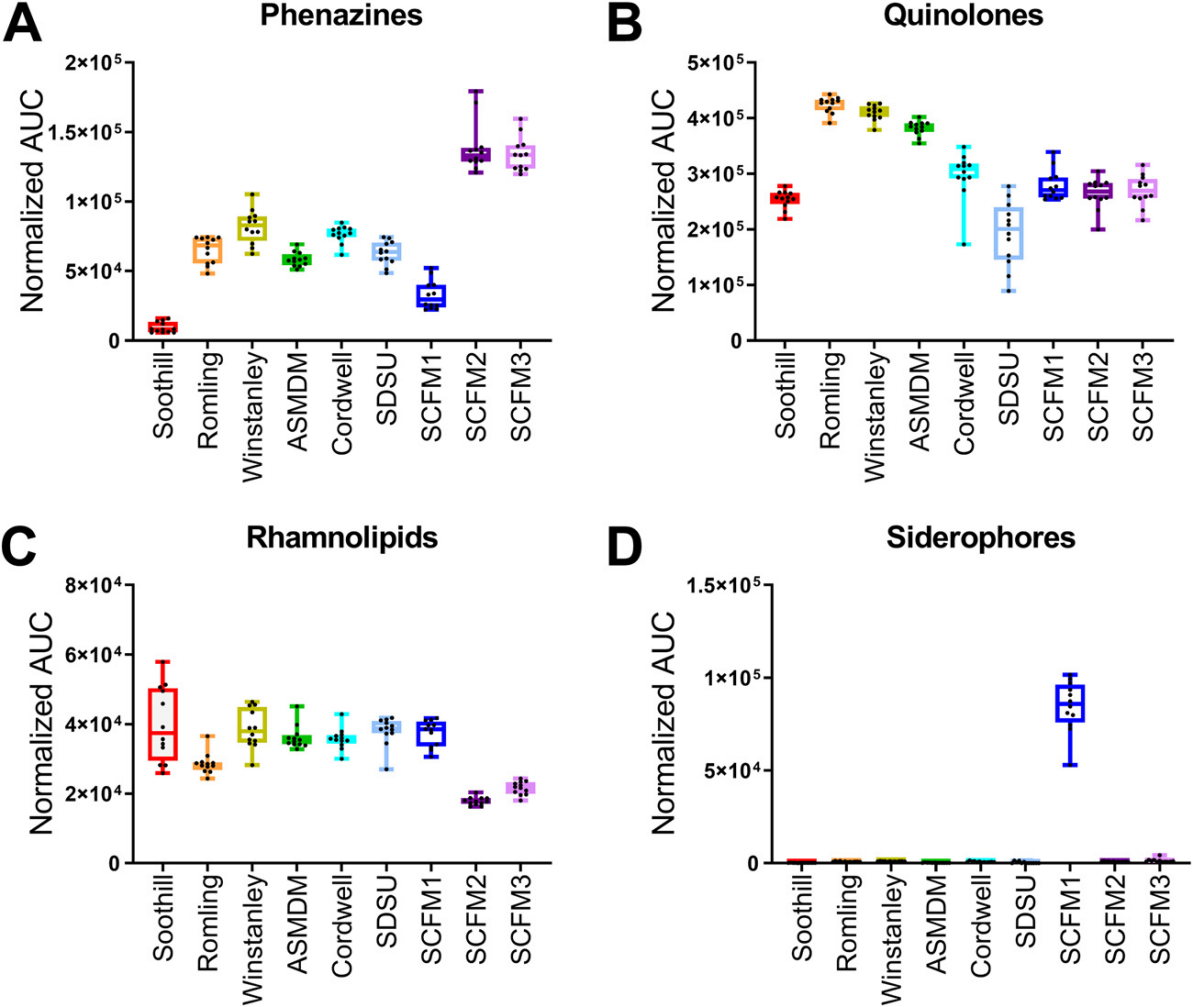
Differential levels of P. aeruginosa secondary metabolite molecular families in ASM. Total normalized area under the curve (AUC) for each secondary metabolite molecular family in each ASM. Box plots represent the 25 to 75th percentiles, with a line at the median. Error bars indicate the minimum to maximum. Individual samples values shown (*n* = 12 biological replicates per ASM formulation). (A) Phenazines. (B) Quinolones. (C) Rhamnolipids. (D) Siderophores, including pyoverdine and pyochelin.

Principal-component analysis identified a small number of highly abundant phenazines and quionolones as differentially produced by PAO1 in different ASM. However, rhamnolipids and the siderophores pyochelin and pyoverdine were also identified in the molecular network. Therefore, to associate these metabolites with nutrient differences between ASM, we performed an analysis of variance (ANOVA) on the feature table. However, the number of features that had statistically different levels between ASM cultures were too numerous to investigate further (Data Set S2). Alternatively, we quantified the total levels of the phenazines, quinolones, rhamnolipids, and siderophores (Fig. 7).

Total phenazine levels were lowest in Soothill ASM samples (Fig. 7A). Proportionally, PCA accounted for ∼90% of the total phenazines produced by PAO1 in Soothill ASM (Fig. S4). All other Soothill-derived ASM had increased levels of phenazines compared to Soothill ASM cultures, with higher proportions of 1-HP and PYO. SCFM1 cultures also had relatively low production of phenazines compared to the other ASM. However, SCFM2 and SCFM3 cultures had higher phenazine production than SCFM1 while maintaining the same percentage of each phenazine.

In all ASM, the quinolones produced by PAO1 were predominantly HHQ type (Fig. S5A), with HHQ, NHQ (C_9_-HQ), and db:NHQ (C_9:1_-HQ) as the most abundant forms (Fig. S5B). Soothill and the SCFM-series ASM cultures had similarly low levels of total quinolones (Fig. 7B). Romling, Winstanley, and ASMDM ASM samples had the highest levels of PQS-type and total quinolones (Fig. S5A). SDSU ASM samples had the lowest average abundance of quinolones, and the variation in total quinolone levels between samples reflects the variability in HQNO production (Fig. 6E). Proportionally, HHQ-type quinolones accounted for 49% (Cordwell ASM) to 76% (Soothill ASM) of total quinolones, followed by HQNO-type (15% [ASMDM ASM] to 41% [SCFM2]), and PQS-type (3% [SCFM1] to 17% [Romling ASM]).

Total rhamnolipid levels were similar in all ASM cultures, except for SCFM2 and SCFM3 (Fig. 7C). PAO1 in SCFM2 and SCFM3 produced approximately 2-fold less rhamnolipids than the SCFM1 cultures. The proportion of mono- to di-rhamnolipids produced by PAO1 was generally maintained across all cultures, ranging from 30% (SDSU ASM) to 44% (Romling ASM) mono-rhamnolipids (Fig. S6A). Similarly, the fatty acids incorporated into rhamnolipids were rather homogeneous across all cultures, with 62% (SDSU ASM) to 72% (Soothill ASM) incorporating C_10_-C_10_ HHAs (Fig. S6B). The other HHAs incorporated include C_10_-C_12:1_ (16% [Soothill ASM] to 22% [Romling ASM]) and C_10_-C_12_ (10% [ASMDM ASM] to 20% [SDSU ASM]).

In the FBMN, the pie chart representation of relative metabolite abundance indicated that pyochelin and pyoverdine levels were highest in SCFM1 cultures. Quantification of total siderophore levels across all ASM samples confirmed this observation (Fig. 7D; Fig. S7). As siderophore biosynthesis is primarily regulated by the bioavailability of environmental iron, this result suggested that SCFM1 was the only iron-limited ASM formulation (43).

### Commercial PGM contains high levels of iron.

**FIG 8:**
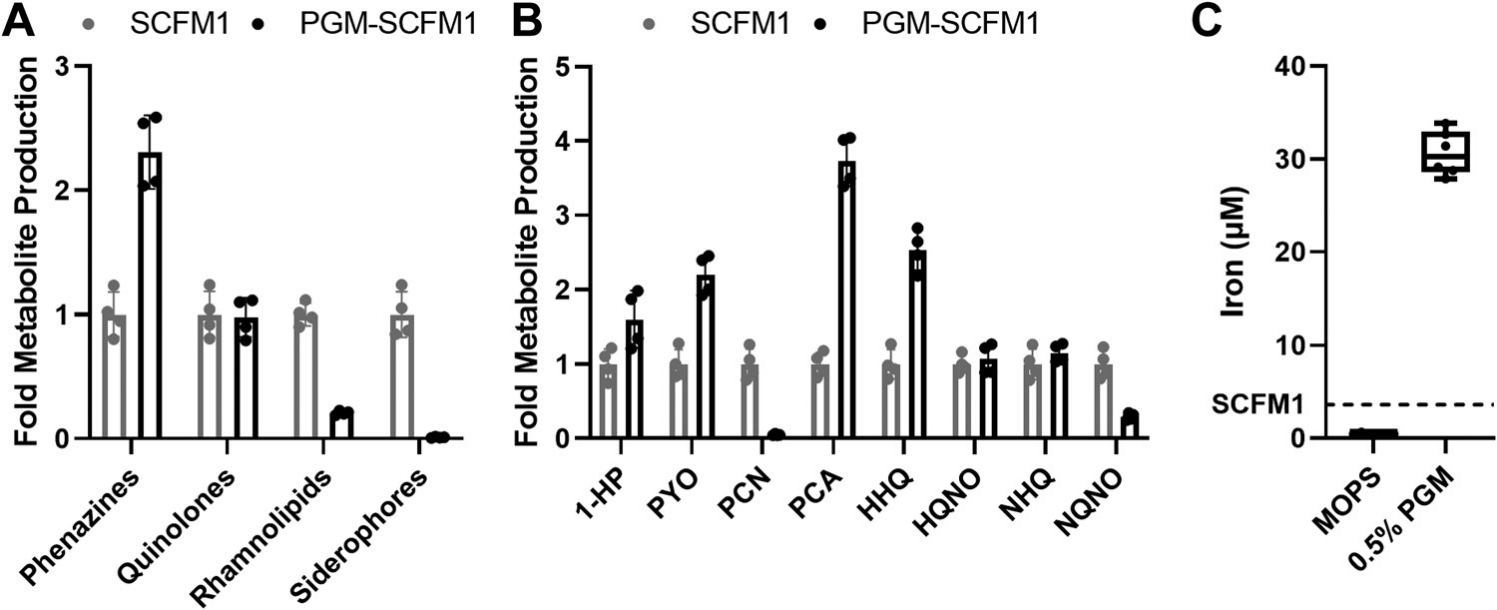
Effect of 0.5% (wt/vol) porcine gastric mucin (PGM) on PAO1 secondary metabolite production. (A) Fold-change difference in production of phenazine, quinolone, rhamnolipid, and siderophore secondary metabolite molecular families by PAO1 between SCFM1 and SCFM1 supplemented with 0.5% PGM (PGM-SCFM1; *n* = 4 biological replicates per medium). (B) Fold-change difference in production of phenazine and select quinolone metabolites by PAO1 between SCFM1 and PGM-SCFM1 (*n* = 4 biological replicates per medium). (C) Concentration of iron in 0.5% PGM and vehicle control (MOPS) (*n* = 6 technical replicates). Dashed line indicates the concentration of iron in SCFM1. 1-HP, 1-hydroxyphenazine; PYO, pyocyanin; PCN, phenazine-1-carboxamide; PCA, phenazine-1-carboxylic acid; HHQ, 2-heptyl-4-quinolone; HQNO, 2-heptyl-4-hydroxyquinoline *N*-oxide; NHQ, 2-nonyl-4-quinolone; NQNO, 2-nonyl-4-hydroxyquinoline *N*-oxide.

We theorized that PGM, the most complex additive of SCFM2 not present in SCFM1, contained a small molecule that was responsible for the differences in PAO1 secondary metabolite production between the cultures. To confirm the effect of PGM on secondary metabolite production, PAO1 was grown in SCFM1 and SCFM1 complemented with 0.5% PGM (PGM-SCFM1). Gross morphology of the biofilms was visualized using a stereomicroscope, and growth was enumerated as CFU per milliliter. Addition of PGM increased the opacity of the cultures and led to a log fold increase of PAO1 growth in PGM-SCFM1 compared to SCFM1 (Fig. S8). P. aeruginosa secondary metabolites were identified and quantified via FBMN (Table S6). The addition of PGM to SCFM1 was sufficient to alter the total levels of the phenazine, rhamnolipid, and siderophore secondary metabolite families (Fig. 8A). PAO1 produced higher levels of total phenazines in PGM-SCFM1 compared to SCFM1, driven by increase in abundance of 1-HP, PYO, and PCA (Fig. 8B). While the total quinolone levels were the same in both PGM-SCFM1 and SCFM1 cultures, the composition of the quinolone molecular family was altered, with an increase in HHQ and decrease in the C_9_ HQNO-like quinolone 2-nonyl-4-hydroxyquinoline *N-*oxide in PGM-SCFM1 samples. Both rhamnolipid and siderophore production by PAO1 was decreased in PGM-SCFM1 compared to SCFM1 cultures.

A mucin suspension was subjected to untargeted LC-MS/MS metabolomics analysis and inductively coupled plasma MS (ICP-MS) to quantify metals. Molecular networking analysis of PGM lipophilic extracts revealed the presence of amino acids, small peptides, and lipids (Data Set S5). ICP-MS analysis revealed that, in addition to other metals (Table S6), a 0.5% (wt/vol) solution of commercial PGM contains approximately 30 μM iron (Fig. 8C). Addition of PGM to SCFM1 increases the total added iron concentration approximately 10-fold.

## DISCUSSION

To study microbial physiology in a CF-like nutrient environment *in vitro*, many ASM have been created with various levels of important nutrients, such as amino acids, mucin, DNA, and iron. (9–20). However, the effect of the different composition of ASM on microbial physiology is poorly understood. Therefore, we applied FBMN to perform comparative analysis of untargeted metabolomics data collected from PAO1 cultured in nine ASM formulations to determine how variations in nutrient composition affect PAO1 secondary metabolite production. Our data indicate a clear relationship between nutrient composition of ASM and virulence-associated metabolite levels produced by PAO1, driven by variation in the concentration of aromatic amino acids and high concentration of iron from the commercial PGM. Importantly, the differences in total and individual secondary metabolite levels produced by P. aeruginosa between ASM influences the ability to compare experimental conclusions, as these metabolites are associated with quorum sensing and virulence.

Phenazines are redox-active metabolites with important roles in electron cycling, oxidative stress, and iron acquisition (44). While 1-HP, PYO, and PCA have been detected in CF sputum samples, their detection has been spurious (29, 45, 46). The low levels of total phenazines produced by PAO1 in Soothill ASM may reflect a shift of chorismate utilization away from phenazine biosynthesis toward the production of aromatic amino acids, as these amino acids are not present in the medium (37). The relatively low level of phenazines produced by PAO1 in Soothill ASM may reflect a reduced virulence phenotype, as highly virulent P. aeruginosa strains have upregulated transcription of genes related to chorismate biosynthesis (47). Phenazine production by PAO1 was highest in SCFM2 and SCFM3. The blue phenotype of these cultures reflected high production of PYO. GlcNAc, a component of SCFM2 and SCFM3, has been shown to increase PYO production by P. aeruginosa, likely through a mechanism to sense and respond to the presence of peptidoglycan (48). Due to the relatively high levels of PYO produced, interactions of P. aeruginosa with lung epithelial cells or other microbes using SCFM2 or SCFM2 as a culture medium may result in increased killing due to the generation of superoxide radicals and increased biofilm formation (49, 50). 1-HP levels were highest in Romling, Winstanley, and ASMDM ASM samples. These ASM contain the highest concentration of total amino acids, which may serve as a preferential carbon source for P. aeruginosa strain PAO1 (51). Catabolism of amino acids to intermediates of the tricarboxylic acid (TCA) cycle may repress transcription of *phzM*, which encodes the methyltransferase required for PYO biosynthesis, and shift phenazine production toward accumulation of 1-HP (37, 52). 1-HP is an integral component of the interkingdom interaction between P. aeruginosa and the fungal CF pathogen Aspergillus fumigatus (40, 53). 1-HP generates reactive oxygen and nitrogen species and contributes to A. fumigatus iron starvation by chelating iron. As a result, A. fumigatus upregulates production of its siderophores to compete for environmental iron. Depending upon the ASM formulation used as culture medium, the 1-HP mediated interaction between P. aeruginosa and A. fumigatus may not be captured. In Soothill ASM, PCA accounted for ∼90% of the phenazines produced, and PCA levels were highest in ASM that contained both amino acids and mucins. The levels of PCA in ASM samples likely reflect an increase in iron uptake via the Feo system, as the primary function of PCA is the reduction of Fe^3+^ to Fe^2+^ (54, 55).

Quinolones produced by P. aeruginosa are involved in biological processes, including quorum sensing, regulation of gene expression for virulence factor production, and microbial competition (39). HHQ, HQNO, and PQS and their C_9_ congeners have been measured from CF samples (29, 56–62). Although PQS is heavily studied, HHQ- and HQNO-type quinolones are the most abundant in CF samples and have been investigated as diagnostic biomarkers of P. aeruginosa infection. Quinolone levels were highest in Romling, Winstanley, and ASMDM ASM samples. These media contain the highest concentrations of aromatic amino acids. The elevated levels of tryptophan in these media increase the available pool of the quinolone precursor anthranilate via the kynurenine pathway, and tyrosine and phenylalanine have been shown to increase quinolone levels by P. aeruginosa in SCFM1 through an undefined mechanism (9, 63, 64). HHQ-type quinolones accounted for ∼90% of the quinolones produced in Soothill ASM cultures, while proportionally lower levels of HHQ-type, but higher levels of PQS-type, quinolones were measured from Romling, Winstanley, and ASMDM ASM samples. HHQ and PQS (and their C_9_ congeners NHQ and C_9_-PQS) activate the quinolone receptor PqsR, driving autoinduction of their biosynthesis and subsequent production of virulence factors associated with the quinolone quorum-sensing pathways (65). Although PQS-type quinolones were the lowest proportion of quinolones produced across all ASM, the PQS levels detected may have a significant impact on virulence factor production by P. aeruginosa. PQS regulates the expression of ∼182 genes, acts as an iron trap when associated with the outer membrane, and promotes the formation of membrane vesicles (66, 67). In ASM formulations that promote PQS biosynthesis, virulence factor production may be elevated and influence the interpretation of related phenotypes. Unlike HHQ and PQS, HQNO-type quinolone production by PAO1 across ASM had no distinct pattern. HQNO affects the growth of the CF opportunistic pathogen Staphylococcus aureus through induction of the small colony variant phenotype, increased biofilm formation, and altering sensitivity to antibiotics (68–70). Therefore, interspecies competition between P. aeruginosa and S. aureus in different ASM may yield different results due to variations in HQNO-type quinolone levels.

Unlike phenazine and quinolone production, rhamnolipid levels were similar between ASM cultures except for those in SCFM2 and SCFM3. Rhamnolipids are virulence-associated glycolipids that function as biosurfactants, which aid in the transport of PQS, development of biofilms, microbial competition, and infiltration of airway epithelial cells by P. aeruginosa (71). A recent study correlated higher levels of rhamnolipid production to virulence phenotypes of 35 P. aeruginosa clinical isolates (72). Two di-rhamnolipids have been consistently detected in CF sputum and explant lung tissue, Rha-Rha-C_10_-C_10_ and Rha-Rha-C_10_-C_12_, although other congeners have been measured (29, 57–60). In ASM, similar di-rhamnolipid production was favored with preferential incorporation of C_10_-C_10_ HAAs. The production of rhamnolipids is regulated by the *rhl* quorum-sensing (QS) system (73). The similar levels of rhamnolipids between most ASM cultures suggest that this QS pathway is not heavily influenced by ASM composition.

The most striking difference in secondary metabolite production by PAO1 among ASM formulations was the production of siderophores. P. aeruginosa produces two siderophores to acquire environmental iron: high-affinity pyoverdine and low-affinity pyochelin. Their production levels are regulated by the ferric uptake regulatory (Fur) system, which senses the amount of bioavailable environmental iron (42, 43). Both siderophores have been measured from CF sputum, although pyoverdine production is strain dependent (58, 59, 74, 75). Pyochelin and pyoverdine were abundant only in SCFM1 cultures. As siderophore production is regulated by the concentration of iron in the medium, the disparate levels of siderophores between SCFM1 and SCFM2 cultures suggest that only SCFM1 was iron limited, although the added iron in the two media was the same.

PGM, the most complex and impure additive of SCFM2, was identified as a source of iron in ASM. Although a variety of CF-relevant nutrients, such as phosphatidylcholines, sphingomyelins, and tryptophan, were detected in PGM, our findings demonstrate that the high concentration of iron (∼30 μM), at least in part, drove changes in levels of PCA, rhamnolipids, and siderophores between SCFM1 and SCFM2 cultures (64, 76, 77). Supporting our conclusion, high concentrations of iron have been shown to repress siderophore production and transcription of rhamnolipid biosynthesis as well as increase PCA levels by P. aeruginosa (43, 54, 78). Importantly, the effect of other components of ASM, including the mucins themselves, may be masked by the impact of the high levels of iron in PGM on P. aeruginosa. We suspect that the concentration of small-molecule components in PGM varies between lots and vendors and may be altered over time by microbial contaminants of the commercial product.

Despite limitations associated with crude commercial mucins, including altered physical and chemical properties due to the purification process and the inclusion of mucin-bound molecules, mucin is a critical medium component for replicating mucosal infection environments *in vitro* (6, 79–81). Of the mucins available to researchers, PGM is the most economic choice. High-purity commercial bovine submaxillary mucins are cost-prohibitive for high-volume or continuous culture studies. Purification of native mucins is both cost- and time-intensive. Only ∼65 mg of PGM can be purified from a single pig stomach (82). To produce 1 liter of ASM containing 0.5% (wt/vol) mucin, at least 77 pig stomachs would be required. Although several groups have established procedures for purifying mucins from commercial preparations, recently developed nonbiological mucin-like polymers show promise toward reflecting the properties of native mucins and may eventually offer a cost-effective alternative (82–87).

While all ASM led to production of secondary metabolites by PAO1, the variations in metabolite levels likely influence the conclusions that can be drawn between experiments performed in ASM of different compositions. To determine associations of nutrient source or concentration to a specific phenotype, SCFM-series ASM containing dialyzed or purified commercial PGM would likely yield the clearest results while remaining economically viable, despite being arduous to prepare. Future modifications of SCFM-series ASM will need to consider nutrients and macromolecules in the CF lung environment that are not present in the current formulations, including bovine serum albumin (BSA), protein-bound iron sources (e.g., ferritin), and bioactive lipids. Additionally, comprehensive characterization of CF samples from patients at different disease stages would support the development of SCFM formulations that emulate the distinct nutrient profiles associated with disease progression and treatment.

## MATERIALS AND METHODS

### Identification of commonly used ASM formulations.

The Web of Science Core Collection (Clarivate Analytics) was searched for papers with the “TOPIC” terms “sputum medium,” “sputum media,” “cystic fibrosis medium,” “cystic fibrosis media,” “sputa medium,” “sputa media,” “CF medium,” or “CF medium” in the title abstract or keyword fields on 10 May 2020. All terms were searched as written, including quotation marks. The results of this search were analyzed using the Web of Science tools “Create Citation Report” and “Analyze Results.” The Analyze Results tool was used to download data corresponding to Web of Science Categories. Google Scholar (Alphabet) was used to quantify the number of citations for the 9 most commonly used ASM formulations (see Table S1 in the supplemental material).

### Preparation of media.

Miller Luria broth (LB; Millipore Sigma) was prepared according to the manufacturer’s instructions. ASM formulations were prepared using the media components, concentrations, and commercial sources in Data Set S1 at the reported pH. ASM were prepared as reported, with the following modifications: no antibiotics were included, 0.1% (wt/vol) agar was excluded from the Cordwell formulation, and porcine gastric mucin type III (PGM; Millipore Sigma) was used in all ASM where mucin was a component (9–20). Prior to use, PGM was suspended in 1× 3-morpholinopropane-1-sulfonic acid (MOPS) buffer (pH 7.0) to make a 10% (wt/vol) suspension and sterilized using a liquid autoclave cycle (<20 min sterilization time), and an aliquot was checked for sterility. PGM was added at the reported final mucin concentration. All media were stored at 4°C, checked for sterility prior to use, and used within 1 month of preparation. PGM-SCFM1 was created by supplementing SCFM1 with 0.5% (wt/vol) PGM.

### Sample preparation of P. aeruginosa growth in ASM and LB.

P. aeruginosa strain PAO1 (MPAO1; University of Washington, Seattle) was inoculated from a streak plate into 5 ml LB and incubated overnight at 37°C with shaking at 220 rpm (88). We inoculated 1.98 ml of LB or ASM with 20 μl of P. aeruginosa LB broth culture (optical density at 600 nm [OD_600_] of 0.05; ∼10^6^ CFU/ml final concentration) in polystyrene 24-well plates. Each medium was evaluated in its own 24-well plate (12 control wells and 12 growth wells per medium). The media were inoculated, disrupted, and extracted in three batches, comprised of 2 to 4 ASM formulations and a parallel LB plate as a control. Batch 1 consisted of LB, Soothill, SDSU, and Cordwell ASM, batch 2 consisted of LB, Romling, Winstanley, ASMDM, and SCFM1 ASM, and batch 3 consisted of LB, SCFM2, and SCFM3 ASM. All plates were covered and incubated statically at 37°C for 72 h. After incubation, gross phenotypes of P. aeruginosa cultures (Fig. 3 and Fig. S1) were photographed using a top-down view with a Stemi 508 stereo microscope with an Axiocam 105 color camera (Zeiss). Following visualization, 100 μl cellulase (Aspergillus niger; 100 mg/ml in sterile water; Millipore Sigma) was added to each well, and aggregates were mechanically disrupted by pipetting using wide-bore pipette tips (USA Scientific), followed by water bath sonication (Branson) for 10 min and a secondary mechanical disruption by pipetting using wide-bore pipette tips. Disrupted samples were aliquoted for measurements of growth (CFU per milliliter and pH [Fig. 3]) and metabolomics analysis. Three representative biological replicates from each culture condition were serially diluted, spotted onto LB agar, incubated at 37°C overnight, and counted to determine CFU per milliliter.

### Sample preparation of P. aeruginosa growth in SCFM1 and PGM-SCFM1.

P. aeruginosa strain PAO1 was inoculated from a streak plate into 5 ml LB and incubated overnight at 37°C with shaking at 220 rpm. We inoculated 1.98 ml of SCFM1 or PGM-SCFM1 (pH 7.0) with 20 μl of P. aeruginosa LB broth culture (OD_600_ of 0.05; ∼10^6^ CFU/ml final concentration) in a polystyrene 24-well plate (88). Both media were evaluated in one 24-well plate (4 control wells and 4 growth wells per medium). The plate was covered and incubated statically at 37°C for 72 h. After incubation, gross phenotypes of P. aeruginosa growth (Fig. S8) were photographed using a top-down view with a Stemi 508 stereo microscope with an Axiocam 105 color camera (Zeiss). Following visualization, aggregates were mechanically disrupted by pipetting using wide-bore pipette tips (USA Scientific). Disrupted samples were aliquoted for measurements of growth (CFU per milliliter and pH; Fig. S8) and metabolomics analysis. All four biological replicates from each culture condition were serially diluted, spotted onto LB agar, incubated at 37°C overnight, and counted to determine CFU per milliliter.

### Metabolomics sample preparation.

HiPerSolv Chromanorm-grade ethyl acetate (EtOAc; VWR) and Optima-grade methanol (MeOH; Fisher Scientific) were used. Each sample aliquot was chemically disrupted with an equal volume of 1:1 EtOAc/MeOH. The samples were dried, resuspended in 100% MeOH containing 1 μM glycocholic acid (Calbiochem; 100.1% pure), diluted as needed in 100% MeOH containing 1 μM glycocholic acid, and centrifuged for 10 min at 4,000 rpm (Thermo; Sorvall ST 40R) to remove nonsoluble particulates. ASM samples were diluted as follows: Soothill, not diluted; Romling, Cordwell, SCFM1, SCFM2, and SCFM3, 5-fold dilution; and Winstanley, ASMDM, SDSU, and LB, 10-fold dilution. SCFM1 and PGM-SCFM1 were diluted 10-fold. Fifty microliters of 10% (wt/vol) PGM in 1× MOPS (morpholinepropanesulfonic acid) was chemically extracted in technical triplicate with 200 μl of 1:1 EtOAc/MeOH following the Bligh-Dyer extraction protocol. The samples were dried and resuspended in 100% MeOH containing 1 μM glycocholic acid, centrifuged for 10 min at 4,000 rpm, and diluted 10-fold.

### Metabolomics data collection.

Mass spectrometry data acquisition for all samples was performed using a Bruker Daltonics Maxis II HD quadrupole time of flight (qTOF) mass spectrometer equipped with a standard electrospray ionization (ESI) source. The mass spectrometer was tuned by infusion of tuning mix ESI-TOF (Agilent Technologies) at a 3-μl/min flow rate. For accurate mass measurements, a wick saturated with hexakis (1H,1H,2H-difluoroethoxy)phosphazene ions (Apollo Scientific, *m/z* 622.1978) located within the source was used as a lock mass internal calibrant. Samples were introduced by an Agilent 1290 ultraperformance liquid chromatography (UPLC) system using a 10-μl injection volume for ASM samples and a 5-μl injection volume for PGM samples. Optima-grade (Fisher Scientific) acetonitrile (ACN), formic acid (FA), and water were used for UPLC separation. Extracts were separated using a Phenomenex Kinetex 2.6-μm C_18_ column (2.1 mm by 50 mm) using a 9 min, linear water-ACN gradient (from 98:2 to 2:98% water/ACN) containing 0.1% FA at a flow rate of 0.5 ml/min. The mass spectrometer was operated in data-dependent positive ion mode, automatically switching between full-scan MS and MS/MS acquisitions. Full-scan MS spectra (*m/z* 50 to 1,500) were acquired in the TOF-MS, and the top five most intense ions in a particular scan were fragmented via collision-induced dissociation (CID) using the stepping function in the collision cell. Data for PA mix, a set of P. aeruginosa secondary metabolite external standards, were acquired under identical conditions. PA mix contains 10 μM each of the following: 1-hydroxyphenazine (1-HP; Tokyo Chemical Industry), pyocyanin (PYO; Sigma), phenazine-1-carboxylic acid (PCA; Ark Pharm), phenazine-1-carboxamide (PCN; Ark Pharm), 2-heptylquinolin-4(1H)-1 (HHQ; Ark Pharm), 2-heptyl-4-hydroxyquinoline *N*-oxide (HQNO, Cayman Chemical), 2-heptyl-3-hydroxyl-4-quinolone (Pseudomonas quinolone signal [PQS]; Chemodex), *N*-butyryl-l-homoserine lactone (C4-HSL; Cayman Chemical), *N*-(3-oxododecanoyl)-l-homoserine lactone (3-oxo-C12-HSL; Millipore Sigma), and a rhamnolipid mixture (AGAE Technologies; 90% pure).

### FBMN of ASM samples.

Bruker Daltonics CompassXport was used to apply lock mass calibration and convert the liquid chromatography-tandem mass spectrometry (LC-MS/MS) metabolomics data from proprietary format to mzXML format. MZmine (version 2.53) was used to perform feature finding on the mzXML files (89). The output files were a feature quantification table and MS/MS spectral summary (mgf). The feature quantification table of molecular ion abundance across all samples was corrected for sample dilution prior to analysis. The MS/MS spectral summary provided a representative MS/MS spectrum for each molecular ion in the feature quantification table. The Feature Networking workflow (version release 22) was applied to the dilution-corrected feature table, MS/MS spectral summary, and a metadata file using the Global Natural Products Social Molecular Networking (GNPS) analysis platform (35, 36). Briefly, the data were filtered by removing all MS/MS fragment ions ± 17 Da of the precursor mass. MS/MS spectra were window filtered by choosing only the top 6 fragment ions in each ±50-Da window throughout the spectrum. The precursor ion mass tolerance was set to 0.05 Da, and the MS/MS fragment ion tolerance was set to 0.1 Da. A network of nodes connected by edges was then created. Edges were filtered to have a cosine score above 0.75. Further, edges between two nodes were kept in the network only if each of the nodes appeared in each other’s respective top 5 most similar nodes. Finally, the maximum size of a molecular family was set to 50, and the lowest-scoring edges were removed from molecular families until the molecular family was below this threshold. The spectra in the network were then searched against the GNPS spectral libraries. The library spectra were filtered in the same manner as the input data. All matches kept between the network spectra and library spectra were required to have a cosine score above 0.6 and at least 5 matched peaks. Sum normalization and mean aggregation were applied. The molecular network (https://gnps.ucsd.edu/ProteoSAFe/status.jsp?task=baba5df5ab1b437980f81b4e64bcffc0) was visualized using Cytoscape (version 3.7.1) (90).

### FBMN of SCFM1 and PGM SCFM1 samples.

Data were processed as described for ASM samples with the following changes: MS/MS spectra were window filtered by choosing only the top 4 fragment ions in each ±50-Da window throughout the spectrum, precursor ion mass tolerance was set to 0.02 Da; MS/MS fragment ion tolerance was set to 0.02 Da, edges were filtered to have a cosine score above 0.7, the maximum size of a molecular family was set to 100, and matches kept between the network spectra and library spectra were required to have a cosine score above 0.7 and at least 4 matched peaks. The molecular network (https://gnps.ucsd.edu/ProteoSAFe/status.jsp?task=5624bf8b3174483a89cf733d08ce0fff) was visualized using Cytoscape (version 3.7.1) (90).

### Annotation of features.

Identification of features in the molecular network as metabolites was accomplished by comparing the experimental data (exact mass, MS/MS fragmentation, and retention time) to (i) the data acquired for the PA Mix standards (level 1 annotation), (ii) reported structural data for features with MS/MS spectral matches to the GNPS libraries (level 2 annotation), and (iii) putative structures for features within in a molecular family (sets of MS/MS spectra from structurally similar molecules). Benzethonium was identified by manually matching the experimental MS/MS data to the data in the Metlin metabolite database (91). Identified features are listed in Table S3 (ASM samples) and Table S5 (SCFM1 and PGM-SCFM1).

### Annotation of metabolites, substructure class, and molecular families.

Multiple features (different adduct ions, in-source fragments, and/or in-source dimers) can represent the same metabolite. Therefore, features associated with the same metabolite were identified by coelution and mass defect from predicted exact mass of the compound adduct, fragment, and/or dimer. Abundance values for features from the same metabolite were summed to provide the total abundance for a metabolite. Abundance values for metabolites with characteristic MS/MS fragmentation patterns were summed to provide total abundance of substructure classes (e.g., HQNO-type quinolones or C_10_-C_10_-containing rhamnolipids). Abundance values for all identified metabolites that were structurally related were summed to provide the total abundance for a molecular family (e.g., quinolones or rhamnolipids). Identified features are listed in Data Set S3 (ASM samples) and Data Set S4 (SCFM1 and PGM-SCFM1).

### Metal quantification from PGM.

We diluted 2.5% (wt/vol) PGM suspended in 1× MOPS 1:1 with nitric acid, and it was then digested overnight and diluted 10-fold. Gallium (Ga), 50 ppb, was added to each sample as an internal standard. Metal concentration was measured by inductively coupled plasma mass spectrometry (ICP-MS) analysis on an Agilent 7500cx at the University of Nebraska-Lincoln Spectroscopy and Biophysics Core facility. Samples were analyzed in technical triplicate with a blank wash run between each sample. Only concentrations above the limit of quantification were retained.

### Classical molecular networking of PGM extracts.

Bruker Daltonics CompassXport was used to apply lock mass calibration and convert the metabolomics data from proprietary format to mzXML format. A classical molecular network was created from the mzXML files using the Molecular Networking workflow (version release 27) of the GNPS web platform (36). Briefly, the data were filtered by removing all MS/MS fragment ions ±17 Da of the precursor mass. MS/MS spectra were window filtered by choosing only the top 6 fragment ions in each ±50-Da window throughout the spectrum. The precursor ion mass tolerance was set to 0.05 Da and the MS/MS fragment ion tolerance to 0.5 Da. A network was then created where edges were filtered to have a cosine score above 0.7 and at least 4 matched fragment peaks between corresponding nodes. Further, edges between two nodes were kept in the network only if each of the nodes appeared in each other’s respective top 10 most similar nodes. Finally, the maximum size of a molecular family was set to 100, and the lowest-scoring edges were removed from molecular families until the molecular family was below this threshold. The spectra in the network were then searched against the GNPS spectral libraries. The library spectra were filtered in the same manner as the input data. All matches kept between the network spectra and library spectra were required to have a cosine score above 0.6 and at least 4 matched peaks. The molecular network (https://gnps.ucsd.edu/ProteoSAFe/status.jsp?task=fc3ee81541aa4a60af38b15f08789620) was visualized in Cytoscape (version 3.7.1) (90). Spectral matches to the GNPS libraries were manually verified by comparing the experimental data (exact mass, MS/MS fragmentation) to reported structures (Data Set S5).

### Statistical analysis.

Statistical comparison of molecular ion abundance across all ASM was conducted on the normalized feature table generated during FBMN analysis in MetaboAnalyst 5.0 using ANOVA with Tukey’s correction for multiple comparisons (92). Statistical comparison of metabolite, molecular family, and substructure class abundance across all ASM was conducted in GraphPad Prism (version 8.4.0) using ANOVA with Tukey’s correction for multiple comparisons. For all analyses, *P* values of <0.05 were considered statistically significant. All statistical results are summarized in Data Set S2.

### Data availability.

All mass spectrometry data, including the raw files, mzXML files, metadata tables, MZmine settings, and Cytoscape files are available via MassIVE as follows: MSV000086721 (LB and ASM formulations media control, P. aeruginosa culture, quality control [QC], and *P. aeruginosa* [PA] standard mix data), MSV000086723 (SCFM1 and PGM-SCFM1 media control, P. aeruginosa culture, QC, and PA mix data), and MSV000087153 (PGM lipophilic extracts).

## References

[1] Kreda SM, Davis CW, Rose MC. 2012. CFTR, mucins, and mucus obstruction in cystic fibrosis. Cold Spring Harb Perspect Med 2:a009589. 10.1101/cshperspect.a009589. 22951447 PMC3426818

[2] Cystic Fibrosis Foundation. 2019. Annual data report. Cystic Fibrosis Foundation, Bethesda, MD.

[3] Konstan MW, Wagener JS, VanDevanter DR, Pasta DJ, Yegin A, Rasouliyan L, Morgan WJ. 2012. Risk factors for rate of decline in FEV1 in adults with cystic fibrosis. J Cystic Fibrosis 11:405–411. 10.1016/j.jcf.2012.03.009. PMC4086189 22561369

[4] Ciofu O, Tolker-Nielsen T, Jensen PØ, Wang H, Høiby N. 2015. Antimicrobial resistance, respiratory tract infections and role of biofilms in lung infections in cystic fibrosis patients. Adv Drug Deliv Rev 85:7–23. 10.1016/j.addr.2014.11.017. 25477303

[5] Sanders NN, Van Rompaey E, De Smedt SC, Demeester J. 2001. Structural alterations of gene complexes by cystic fibrosis sputum. Am J Respir Crit Care Med 164:486–493. 10.1164/ajrccm.164.3.2011041. 11500355

[6] Schwab U, Abdullah LH, Perlmutt OS, Albert D, Davis CW, Arnold RR, Yankaskas JR, Gilligan P, Neubauer H, Randell SH, Boucher RC. 2014. Localization of Burkholderia cepacia complex bacteria in cystic fibrosis lungs and interactions with Pseudomonas aeruginosa in hypoxic mucus. Infect Immun 82:4729–4745. 10.1128/IAI.01876-14. 25156735 PMC4249344

[7] Wolak JE, Esther CR, Jr., O'Connell TM. 2009. Metabolomic analysis of bronchoalveolar lavage fluid from cystic fibrosis patients. Biomarkers 14:55–60. 10.1080/13547500802688194. 19283525 PMC3837581

[8] La Rosa R, Johansen HK, Molin S. 2019. Adapting to the airways: metabolic Requirements of Pseudomonas aeruginosa during the infection of cystic fibrosis patients. Metabolites 9:234. 10.3390/metabo9100234. PMC6835255 31623245

[9] Palmer KL, Aye LA, Whiteley M. 2007. Nutritional cues control Pseudomonas aeruginosa multicellular behavior in cystic fibrosis sputum. J Bacteriol 189:8079–8087. 10.1128/JB.01138-07. 17873029 PMC2168676

[10] Ghani M, Soothill JS. 1997. Ceftazidime, gentamicin, and rifampicin, in combination, kill biofilms of mucoid Pseudomonas aeruginosa. Can J Microbiol 43:999–1004. 10.1139/m97-144. 9436304

[11] Diraviam Dinesh S, Diraviam Dinesh S. 2010. Artifical sputum medium. Protoc Exch 10.1038/protex.2010.212.

[12] Sriramulu DD, Lunsdorf H, Lam JS, Romling U. 2005. Microcolony formation: a novel biofilm model of Pseudomonas aeruginosa for the cystic fibrosis lung. J Med Microbiol 54:667–676. 10.1099/jmm.0.45969-0. 15947432

[13] Fung C, Naughton S, Turnbull L, Tingpej P, Rose B, Arthur J, Hu HH, Harmer C, Harbour C, Hassett DJ, Whitchurch CB, Manos J. 2010. Gene expression of Pseudomonas aeruginosa in a mucin-containing synthetic growth medium mimicking cystic fibrosis lung sputum. J Med Microbiol 59:1089–1100. 10.1099/jmm.0.019984-0. 20522626

[14] Hare NJ, Soe CZ, Rose B, Harbour C, Codd R, Manos J, Cordwell SJ. 2012. Proteomics of Pseudomonas aeruginosa Australian epidemic strain 1 (AES-1) cultured under conditions mimicking the cystic fibrosis lung reveals increased iron acquisition via the siderophore pyochelin. J Proteome Res 11:776–795. 10.1021/pr200659h. 22054071

[15] Kirchner S, Fothergill JL, Wright EA, James CE, Mowat E, Winstanley C. 2012. Use of artificial sputum medium to test antibiotic efficacy against Pseudomonas aeruginosa in conditions more relevant to the cystic fibrosis lung. J Vis Exp e3857. 10.3791/3857. 22711026 PMC3471314

[16] Quinn RA, Whiteson K, Lim YW, Salamon P, Bailey B, Mienardi S, Sanchez SE, Blake D, Conrad D, Rohwer F. 2015. A Winogradsky-based culture system shows an association between microbial fermentation and cystic fibrosis exacerbation. ISME J 9:1024–1038. 10.1038/ismej.2014.234. 25514533 PMC4817692

[17] Turner KH, Wessel AK, Palmer GC, Murray JL, Whiteley M. 2015. Essential genome of Pseudomonas aeruginosa in cystic fibrosis sputum. Proc Natl Acad Sci USA 112:4110–4115. 10.1073/pnas.1419677112. 25775563 PMC4386324

[18] Wong A, Rodrigue N, Kassen R. 2012. Genomics of adaptation during experimental evolution of the opportunistic pathogen Pseudomonas aeruginosa. PLoS Genet 8:e1002928. 10.1371/journal.pgen.1002928. 23028345 PMC3441735

[19] Schick A, Kassen R. 2018. Rapid diversification of Pseudomonas aeruginosa in cystic fibrosis lung-like conditions. Proc Natl Acad Sci USA 115:10714–10719. 10.1073/pnas.1721270115. 30275334 PMC6196507

[20] Homa M, Sandor A, Toth E, Szebenyi C, Nagy G, Vagvolgyi C, Papp T. 2019. In vitro interactions of Pseudomonas aeruginosa with Scedosporium species frequently associated with cystic fibrosis. Front Microbiol 10:441. 10.3389/fmicb.2019.00441. 30894846 PMC6414507

[21] Behrends V, Ebbels TMD, Williams HD, Bundy JG. 2009. Time-resolved metabolic footprinting for nonlinear modeling of bacterial substrate utilization. Appl Environ Microbiol 75:2453–2463. 10.1128/AEM.01742-08. 19218401 PMC2675220

[22] Behrends V, Geier B, Williams HD, Bundy JG. 2013. Direct assessment of metabolite utilization by Pseudomonas aeruginosa during growth on artificial sputum medium. Appl Environ Microbiol 79:2467–2470. 10.1128/AEM.03609-12. 23354718 PMC3623229

[23] Darch SE, Simoska O, Fitzpatrick M, Barraza JP, Stevenson KJ, Bonnecaze RT, Shear JB, Whiteley M. 2018. Spatial determinants of quorum signaling in a Pseudomonas aeruginosa infection model. Proc Natl Acad Sci USA 115:4779–4784. 10.1073/pnas.1719317115. 29666244 PMC5939081

[24] Diaz Iglesias Y, Van Bambeke F. 2020. Activity of antibiotics against Pseudomonas aeruginosa in an in vitro model of biofilms in the context of cystic fibrosis: influence of the culture medium. Antimicrob Agents Chemother 64:e02204-19. 10.1128/AAC.02204-19. 32015047 PMC7179293

[25] Haley CL, Colmer-Hamood JA, Hamood AN. 2012. Characterization of biofilm-like structures formed by Pseudomonas aeruginosa in a synthetic mucus medium. BMC Microbiol 12:181. 10.1186/1471-2180-12-181. 22900764 PMC3494610

[26] Kirchhoff L, Weisner A-K, Schrepffer M, Hain A, Scharmann U, Buer J, Rath P-M, Steinmann J. 2020. Phenotypical characteristics of the black yeast Exophiala dermatitidis are affected by Pseudomonas aeruginosa in an artificial sputum medium mimicking cystic fibrosis–like conditions. Front Microbiol 11:471. 10.3389/fmicb.2020.00471. 32265891 PMC7100538

[27] Lightly TJ, Phung RR, Sorensen JL, Cardona ST. 2017. Synthetic cystic fibrosis sputum medium diminishes Burkholderia cenocepacia antifungal activity against Aspergillus fumigatus independently of phenylacetic acid production. Can J Microbiol 63:427–438. 10.1139/cjm-2016-0705. 28178425

[28] Limoli DH, Whitfield GB, Kitao T, Ivey ML, Davis MR, Grahl N, Hogan DA, Rahme LG, Howell PL, O’Toole GA, Goldberg JB. 2017. Pseudom00186-17onas aeruginosa alginate overproduction promotes coexistence with Staphylococcus aureus in a model of cystic fibrosis respiratory infection. mBio 8:e00186-17. 10.1128/mBio.00186-17. 28325763 PMC5362032

[29] Quinn RA, Phelan VV, Whiteson KL, Garg N, Bailey BA, Lim YW, Conrad DJ, Dorrestein PC, Rohwer FL. 2016. Microbial, host and xenobiotic diversity in the cystic fibrosis sputum metabolome. ISME J 10:1483–1498. 10.1038/ismej.2015.207. 26623545 PMC5029181

[30] Stevens DA, Moss RB, Hernandez C, Clemons KV, Martinez M. 2016. Effect of media modified to mimic cystic fibrosis sputum on the susceptibility of Aspergillus fumigatus, and the frequency of resistance at one center. Antimicrob Agents Chemother 60:2180–2184. 10.1128/AAC.02649-15. 26810647 PMC4808157

[31] Jurado-Martin I, Sainz-Mejias M, McClean S. 2021. Pseudomonas aeruginosa: an audacious pathogen with an adaptable arsenal of virulence Factors. Int J Mol Sci 22:3128. 10.3390/ijms22063128. 33803907 PMC8003266

[32] Huang W, Brewer LK, Jones JW, Nguyen AT, Marcu A, Wishart DS, Oglesby-Sherrouse AG, Kane MA, Wilks A. 2018. PAMDB: a comprehensive Pseudomonas aeruginosa metabolome database. Nucleic Acids Res 46:D575–D580. 10.1093/nar/gkx1061. 29106626 PMC5753269

[33] Phelan VV, Moree WJ, Aguilar J, Cornett DS, Koumoutsi A, Noble SM, Pogliano K, Guerrero CA, Dorrestein PC. 2014. Impact of a transposon insertion in phzF2 on the specialized metabolite production and interkingdom interactions of Pseudomonas aeruginosa. J Bacteriol 196:1683–1693. 10.1128/JB.01258-13. 24532776 PMC3993319

[34] Quinn RA, Comstock W, Zhang T, Morton JT, da Silva R, Tran A, Aksenov A, Nothias LF, Wangpraseurt D, Melnik AV, Ackermann G, Conrad D, Klapper I, Knight R, Dorrestein PC. 2018. Niche partitioning of a pathogenic microbiome driven by chemical gradients. Sci Adv 4:eaau1908. 10.1126/sciadv.aau1908. 30263961 PMC6157970

[35] Nothias L-F, Petras D, Schmid R, Dührkop K, Rainer J, Sarvepalli A, Protsyuk I, Ernst M, Tsugawa H, Fleischauer M, Aicheler F, Aksenov AA, Alka O, Allard P-M, Barsch A, Cachet X, Caraballo-Rodriguez AM, Da Silva RR, Dang T, Garg N, Gauglitz JM, Gurevich A, Isaac G, Jarmusch AK, Kameník Z, Kang KB, Kessler N, Koester I, Korf A, Le Gouellec A, Ludwig M, Martin H C, McCall L-I, McSayles J, Meyer SW, Mohimani H, Morsy M, Moyne O, Neumann S, Neuweger H, Nguyen NH, Nothias-Esposito M, Paolini J, Phelan VV, Pluskal T, Quinn RA, Rogers S, Shrestha B, Tripathi A, van der Hooft JJJ, et al. 2020. Feature-based molecular networking in the GNPS analysis environment. Nat Methods 17:905–908. 10.1038/s41592-020-0933-6. 32839597 PMC7885687

[36] Wang M, Carver JJ, Phelan VV, Sanchez LM, Garg N, Peng Y, Nguyen DD, Watrous J, Kapono CA, Luzzatto-Knaan T, Porto C, Bouslimani A, Melnik AV, Meehan MJ, Liu WT, Crusemann M, Boudreau PD, Esquenazi E, Sandoval-Calderon M, Kersten RD, Pace LA, Quinn RA, Duncan KR, Hsu CC, Floros DJ, Gavilan RG, Kleigrewe K, Northen T, Dutton RJ, Parrot D, Carlson EE, Aigle B, Michelsen CF, Jelsbak L, Sohlenkamp C, Pevzner P, Edlund A, McLean J, Piel J, Murphy BT, Gerwick L, Liaw CC, Yang YL, Humpf HU, Maansson M, Keyzers RA, Sims AC, Johnson AR, Sidebottom AM, Sedio BE, et al. 2016. Sharing and community curation of mass spectrometry data with Global Natural Products Social Molecular Networking. Nat Biotechnol 34:828–837. 10.1038/nbt.3597. 27504778 PMC5321674

[37] Mentel M, Ahuja EG, Mavrodi DV, Breinbauer R, Thomashow LS, Blankenfeldt W. 2009. Of two make one: the biosynthesis of phenazines. Chembiochem 10:2295–2304. 10.1002/cbic.200900323. 19658148

[38] Lepine F, Milot S, Deziel E, He J, Rahme LG. 2004. Electrospray/mass spectrometric identification and analysis of 4-hydroxy-2-alkylquinolines (HAQs) produced by Pseudomonas aeruginosa. J Am Soc Mass Spectrom 15:862–869. 10.1016/j.jasms.2004.02.012. 15144975

[39] Heeb S, Fletcher MP, Chhabra SR, Diggle SP, Williams P, Camara M. 2011. Quinolones: from antibiotics to autoinducers. FEMS Microbiol Rev 35:247–274. 10.1111/j.1574-6976.2010.00247.x. 20738404 PMC3053476

[40] Moree WJ, Phelan VV, Wu C-H, Bandeira N, Cornett DS, Duggan BM, Dorrestein PC. 2012. Interkingdom metabolic transformations captured by microbial imaging mass spectrometry. Proc Natl Acad Sci USA 109:13811–13816. 10.1073/pnas.1206855109. 22869730 PMC3427086

[41] Deziel E, Lepine F, Milot S, Villemur R. 2003. rhlA is required for the production of a novel biosurfactant promoting swarming motility in Pseudomonas aeruginosa: 3-(3-hydroxyalkanoyloxy)alkanoic acids (HAAs), the precursors of rhamnolipids. Microbiology (Reading) 149:2005–2013. 10.1099/mic.0.26154-0. 12904540

[42] Ringel MT, Brüser T. 2018. The biosynthesis of pyoverdines. Microb Cell 5:424–437. 10.15698/mic2018.10.649. 30386787 PMC6206403

[43] Cornelis P, Dingemans J. 2013. Pseudomonas aeruginosa adapts its iron uptake strategies in function of the type of infections. Front Cell Infect Microbiol 3:75. 10.3389/fcimb.2013.00075. 24294593 PMC3827675

[44] Blankenfeldt W, Parsons JF. 2014. The structural biology of phenazine biosynthesis. Curr Opin Struct Biol 29:26–33. 10.1016/j.sbi.2014.08.013. 25215885 PMC4268259

[45] Wilson R, Sykes DA, Watson D, Rutman A, Taylor GW, Cole PJ. 1988. Measurement of Pseudomonas aeruginosa phenazine pigments in sputum and assessment of their contribution to sputum sol toxicity for respiratory epithelium. Infect Immun 56:2515–2517. 10.1128/iai.56.9.2515-2517.1988. 3137173 PMC259599

[46] Glasser NR, Hunter RC, Liou TG, Newman DK, Mountain West CFCI, for the Mountain West CF Consortium Investigators. 2019. Refinement of metabolite detection in cystic fibrosis sputum reveals heme correlates with lung function decline. PLoS One 14:e0226578. 10.1371/journal.pone.0226578. 31851705 PMC6919587

[47] Panayidou S, Georgiades K, Christofi T, Tamana S, Promponas VJ, Apidianakis Y. 2020. Pseudomonas aeruginosa core metabolism exerts a widespread growth-independent control on virulence. Sci Rep 10:9505. 10.1038/s41598-020-66194-4. 32528034 PMC7289854

[48] Korgaonkar AK, Whiteley M. 2011. Pseudomonas aeruginosa enhances production of an antimicrobial in response to N-acetylglucosamine and peptidoglycan. J Bacteriol 193:909–917. 10.1128/JB.01175-10. 21169497 PMC3028681

[49] Das T, Manefield M. 2013. Phenazine production enhances extracellular DNA release via hydrogen peroxide generation in Pseudomonas aeruginosa. Commun Integr Biol 6:e23570. 10.4161/cib.23570. 23710274 PMC3656008

[50] Das T, Manefield M. 2012. Pyocyanin promotes extracellular DNA release in Pseudomonas aeruginosa. PLoS One 7:e46718. 10.1371/journal.pone.0046718. 23056420 PMC3466280

[51] Dolan SK, Kohlstedt M, Trigg S, Vallejo Ramirez P, Kaminski CF, Wittmann C, Welch M. 2020. Contextual flexibility in Pseudomonas aeruginosa central carbon metabolism during growth in single carbon sources. mBio 11:e02684-19. 10.1128/mBio.02684-19. 32184246 PMC7078475

[52] Huang JF, Sonnleitner E, Ren B, Xu YQ, Haas D. 2012. Catabolite repression control of pyocyanin biosynthesis at an intersection of primary and secondary metabolism in Pseudomonas aeruginosa. Appl Environ Microbiol 78:5016–5020. 10.1128/AEM.00026-12. 22562990 PMC3416368

[53] Briard B, Bomme P, Lechner BE, Mislin GL, Lair V, Prevost MC, Latge JP, Haas H, Beauvais A. 2015. Pseudomonas aeruginosa manipulates redox and iron homeostasis of its microbiota partner Aspergillus fumigatus via phenazines. Sci Rep 5:8220. 10.1038/srep08220. 25665925 PMC5389140

[54] Wang Y, Wilks JC, Danhorn T, Ramos I, Croal L, Newman DK. 2011. Phenazine-1-carboxylic acid promotes bacterial biofilm development via ferrous iron acquisition. J Bacteriol 193:3606–3617. 10.1128/JB.00396-11. 21602354 PMC3133341

[55] Cartron ML, Maddocks S, Gillingham P, Craven CJ, Andrews SC. 2006. Feo - transport of ferrous iron into bacteria. Biometals 19:143–157. 10.1007/s10534-006-0003-2. 16718600

[56] Barr HL, Halliday N, Barrett DA, Williams P, Forrester DL, Peckham D, Williams K, Smyth AR, Honeybourne D, Whitehouse JL, Nash EF, Dewar J, Clayton A, Knox AJ, Camara M, Fogarty AW. 2017. Diagnostic and prognostic significance of systemic alkyl quinolones for P aeruginosa in cystic fibrosis: a longitudinal study. J Cyst Fibros 16:230–238. 10.1016/j.jcf.2016.10.005. 27773591 PMC5345566

[57] Melnik AV, Vazquez-Baeza Y, Aksenov AA, Hyde E, McAvoy AC, Wang M, da Silva RR, Protsyuk I, Wu JV, Bouslimani A, Lim YW, Luzzatto-Knaan T, Comstock W, Quinn RA, Wong R, Humphrey G, Ackermann G, Spivey T, Brouha SS, Bandeira N, Lin GY, Rohwer F, Conrad DJ, Alexandrov T, Knight R, Dorrestein PC, Garg N. 2019. Molecular and microbial microenvironments in chronically diseased lungs associated with cystic fibrosis. mSystems 4:e00375-19. 10.1128/mSystems.00375-19. 31551401 PMC6759567

[58] Quinn RA, Adem S, Mills RH, Comstock W, DeRight Goldasich L, Humphrey G, Aksenov AA, Melnik AV, da Silva R, Ackermann G, Bandeira N, Gonzalez DJ, Conrad D, O'Donoghue AJ, Knight R, Dorrestein PC. 2019. Neutrophilic proteolysis in the cystic fibrosis lung correlates with a pathogenic microbiome. Microbiome 7:23. 10.1186/s40168-019-0636-3. 30760325 PMC6375204

[59] Raghuvanshi R, Vasco K, Vazquez-Baeza Y, Jiang L, Morton JT, Li D, Gonzalez A, DeRight Goldasich L, Humphrey G, Ackermann G, Swafford AD, Conrad D, Knight R, Dorrestein PC, Quinn RA. 2020. High-resolution longitudinal dynamics of the cystic fibrosis sputum microbiome and metabolome through antibiotic therapy. mSystems 5:e00292-20. 10.1128/mSystems.00292-20. 32576651 PMC7311317

[60] Garg N, Wang M, Hyde E, da Silva RR, Melnik AV, Protsyuk I, Bouslimani A, Lim YW, Wong R, Humphrey G, Ackermann G, Spivey T, Brouha SS, Bandeira N, Lin GY, Rohwer F, Conrad DJ, Alexandrov T, Knight R, Dorrestein PC. 2017. Three-dimensional microbiome and metabolome cartography of a diseased human lung. Cell Host Microbe 22:705–716.e4. 10.1016/j.chom.2017.10.001. 29056429 PMC6267898

[61] Collier DN, Anderson L, McKnight SL, Noah TL, Knowles M, Boucher R, Schwab U, Gilligan P, Pesci EC. 2002. A bacterial cell to cell signal in the lungs of cystic fibrosis patients. FEMS Microbiol Lett 215:41–46. 10.1111/j.1574-6968.2002.tb11367.x. 12393198

[62] Webb K, Fogarty A, Barrett DA, Nash EF, Whitehouse JL, Smyth AR, Stewart I, Knox A, Williams P, Halliday N, Camara M, Barr HL. 2019. Clinical significance of Pseudomonas aeruginosa 2-alkyl-4-quinolone quorum-sensing signal molecules for long-term outcomes in adults with cystic fibrosis. J Med Microbiol 68:1823–1828. 10.1099/jmm.0.001099. 31671047

[63] Palmer KL, Mashburn LM, Singh PK, Whiteley M. 2005. Cystic fibrosis sputum supports growth and cues key aspects of Pseudomonas aeruginosa physiology. J Bacteriol 187:5267–5277. 10.1128/JB.187.15.5267-5277.2005. 16030221 PMC1196007

[64] Bortolotti P, Hennart B, Thieffry C, Jausions G, Faure E, Grandjean T, Thepaut M, Dessein R, Allorge D, Guery BP, Faure K, Kipnis E, Toussaint B, Le Gouellec A. 2016. Tryptophan catabolism in Pseudomonas aeruginosa and potential for inter-kingdom relationship. BMC Microbiol 16:137. 10.1186/s12866-016-0756-x. 27392067 PMC4938989

[65] Ilangovan A, Fletcher M, Rampioni G, Pustelny C, Rumbaugh K, Heeb S, Camara M, Truman A, Chhabra SR, Emsley J, Williams P. 2013. Structural basis for native agonist and synthetic inhibitor recognition by the Pseudomonas aeruginosa quorum sensing regulator PqsR (MvfR). PLoS Pathog 9:e1003508. 10.1371/journal.ppat.1003508. 23935486 PMC3723537

[66] Diggle SP, Matthijs S, Wright VJ, Fletcher MP, Chhabra SR, Lamont IL, Kong X, Hider RC, Cornelis P, Camara M, Williams P. 2007. The Pseudomonas aeruginosa 4-quinolone signal molecules HHQ and PQS play multifunctional roles in quorum sensing and iron entrapment. Chem Biol 14:87–96. 10.1016/j.chembiol.2006.11.014. 17254955

[67] Lin JS, Cheng JL, Wang Y, Shen XH. 2018. The Pseudomonas quinolone signal (PQS): not just for quorum sensing anymore. Front Cell Infect Microbiol 8:230. 10.3389/fcimb.2018.00230. 30023354 PMC6039570

[68] Hazan R, Que YA, Maura D, Strobel B, Majcherczyk PA, Hopper LR, Wilbur DJ, Hreha TN, Barquera B, Rahme LG. 2016. Auto poisoning of the respiratory chain by a quorum-sensing-regulated molecule favors biofilm formation and antibiotic tolerance. Curr Biol 26:195–206. 10.1016/j.cub.2015.11.056. 26776731 PMC4729643

[69] Hoffman LR, Deziel E, D'Argenio DA, Lepine F, Emerson J, McNamara S, Gibson RL, Ramsey BW, Miller SI. 2006. Selection for Staphylococcus aureus small-colony variants due to growth in the presence of Pseudomonas aeruginosa. Proc Natl Acad Sci USA 103:19890–19895. 10.1073/pnas.0606756104. 17172450 PMC1750898

[70] Orazi G, O'Toole GA. 2017. Pseudomonas aeruginosa alters Staphylococcus aureus sensitivity to vancomycin in a biofilm model of cystic fibrosis infection. mBio 8 10.1128/mBio.00873-17. PMC5516255 28720732

[71] Abdel-Mawgoud AM, Lepine F, Deziel E. 2010. Rhamnolipids: diversity of structures, microbial origins and roles. Appl Microbiol Biotechnol 86:1323–1336. 10.1007/s00253-010-2498-2. 20336292 PMC2854365

[72] Depke T, Thoming JG, Kordes A, Haussler S, Bronstrup M. 2020. Untargeted LC-MS metabolomics differentiates between virulent and avirulent clinical strains of Pseudomonas aeruginosa. Biomolecules 10:1041. 10.3390/biom10071041. PMC7407980 32668735

[73] Dusane DH, Zinjarde SS, Venugopalan VP, McLean RJ, Weber MM, Rahman PK. 2010. Quorum sensing: implications on rhamnolipid biosurfactant production. Biotechnol Genet Eng Rev 27:159–184. 10.1080/02648725.2010.10648149. 21415897

[74] Kang D, Revtovich AV, Chen Q, Shah KN, Cannon CL, Kirienko NV. 2019. Pyoverdine-dependent virulence of Pseudomonas aeruginosa isolates from cystic fibrosis patients. Front Microbiol 10:2048. 10.3389/fmicb.2019.02048. 31551982 PMC6743535

[75] Martin LW, Reid DW, Sharples KJ, Lamont IL. 2011. Pseudomonas siderophores in the sputum of patients with cystic fibrosis. Biometals 24:1059–1067. 10.1007/s10534-011-9464-z. 21643731

[76] Bernhard W, Hoffmann S, Dombrowsky H, Rau GA, Kamlage A, Kappler M, Haitsma JJ, Freihorst J, von der Hardt H, Poets CF. 2001. Phosphatidylcholine molecular species in lung surfactant composition in relation to respiratory rate and lung development. Am J Respir Cell Mol Biol 25:725–731. 10.1165/ajrcmb.25.6.4616. 11726398

[77] Aureli M, Schiumarini D, Loberto N, Bassi R, Tamanini A, Mancini G, Tironi M, Munari S, Cabrini G, Dechecchi MC, Sonnino S. 2016. Unravelling the role of sphingolipids in cystic fibrosis lung disease. Chem Phys Lipids 200:94–103. 10.1016/j.chemphyslip.2016.08.002. 27592248

[78] Yu S, Wei Q, Zhao T, Guo Y, Ma LZ. 2016. A survival strategy for Pseudomonas aeruginosa that uses exopolysaccharides to sequester and store iron to stimulate Psl-dependent biofilm formation. Appl Environ Microbiol 82:6403–6413. 10.1128/AEM.01307-16. 27565622 PMC5066357

[79] Flynn JM, Niccum D, Dunitz JM, Hunter RC. 2016. Evidence and role for bacterial mucin degradation in cystic fibrosis airway disease. PLoS Pathog 12:e1005846. 10.1371/journal.ppat.1005846. 27548479 PMC4993466

[80] Hoffman CL, Lalsiamthara J, Aballay A. 2020. Host mucin is exploited by Pseudomonas aeruginosa to provide monosaccharides required for a successful infection. mBio 11:e00060-20. 10.1128/mBio.00060-20. 32127446 PMC7064748

[81] Wheeler KM, Carcamo-Oyarce G, Turner BS, Dellos-Nolan S, Co JY, Lehoux S, Cummings RD, Wozniak DJ, Ribbeck K. 2019. Mucin glycans attenuate the virulence of Pseudomonas aeruginosa in infection. Nat Microbiol 4:2146–2154. 10.1038/s41564-019-0581-8. 31611643 PMC7157942

[82] Schomig VJ, Kasdorf BT, Scholz C, Bidmon K, Lieleg O, Berensmeier S. 2016. An optimized purification process for porcine gastric mucin with preservation of its native functional properties. Rsc Adv 6:44932–44943. 10.1039/C6RA07424C.

[83] Kocevar-Nared J, Kristl J, Smid-Korbar J. 1997. Comparative rheological investigation of crude gastric mucin and natural gastric mucus. Biomaterials 18:677–681. 10.1016/S0142-9612(96)00180-9. 9151999

[84] Lieleg O, Lieleg C, Bloom J, Buck CB, Ribbeck K. 2012. Mucin biopolymers as broad-spectrum antiviral agents. Biomacromolecules 13:1724–1732. 10.1021/bm3001292. 22475261 PMC3597216

[85] Sturmer R, Harder S, Schluter H, Hoffmann W. 2018. Commercial porcine gastric mucin preparations, also used as artificial saliva, are a rich source for the lectin TFF2: in vitro binding studies. Chembiochem 19:2598–2608. 10.1002/cbic.201800622. 30371971

[86] Werlang C, Carcarmo-Oyarce G, Ribbeck K. 2019. Engineering mucus to study and influence the microbiome. Nat Rev Mater 4:134–145. 10.1038/s41578-018-0079-7. PMC11906034 40084234

[87] Kruger AG, Brucks SD, Yan T, Carcarmo-Oyarce G, Wei Y, Wen DH, Carvalho DR, Hore MJA, Ribbeck K, Schrock RR, Kiessling LL. 2021. Stereochemical control yields mucin mimetic polymers. Acs Cent Sci 7:624–630. 10.1021/acscentsci.0c01569. 34056092 PMC8155468

[88] Jacobs MA, Alwood A, Thaipisuttikul I, Spencer D, Haugen E, Ernst S, Will O, Kaul R, Raymond C, Levy R, Liu CR, Guenthner D, Bovee D, Olson MV, Manoil C. 2003. Comprehensive transposon mutant library of Pseudomonas aeruginosa. Proc Natl Acad Sci USA 100:14339–14344. 10.1073/pnas.2036282100. 14617778 PMC283593

[89] Pluskal T, Castillo S, Villar-Briones A, Oresic M. 2010. MZmine 2: modular framework for processing, visualizing, and analyzing mass spectrometry-based molecular profile data. Bmc Bioinformatics 11:395. 10.1186/1471-2105-11-395. 20650010 PMC2918584

[90] Shannon P, Markiel A, Ozier O, Baliga NS, Wang JT, Ramage D, Amin N, Schwikowski B, Ideker T. 2003. Cytoscape: a software environment for integrated models of biomolecular interaction networks. Genome Res 13:2498–2504. 10.1101/gr.1239303. 14597658 PMC403769

[91] Xue JC, Guijas C, Benton HP, Warth B, Siuzdak G. 2020. METLIN MS2 molecular standards database: a broad chemical and biological resource. Nat Methods 17:953–954. 10.1038/s41592-020-0942-5. 32839599 PMC8802982

[92] Pang Z, Chong J, Zhou G, de Lima Morais DA, Chang L, Barrette M, Gauthier C, Jacques PE, Li S, Xia J. 2021. MetaboAnalyst 5.0: narrowing the gap between raw spectra and functional insights. Nucleic Acids Res 49:W388–W396. 10.1093/nar/gkab382. 34019663 PMC8265181

