# Supplementary material for: Impact of Artificial Sputum Medium Formulation on Pseudomonas aeruginosa Secondary Metabolite Production
Source: J Bacteriol. 2021 Oct 12;203(21):e00250-21. doi: 10.1128/JB.00250-21 (PMC8508215; doi:10.1128/JB.00250-21)
Supplement: Supplemental file 6 — Tables S1 to S6 and Fig. S1 to S8. Download JB.00250-21-s0001.pdf, PDF file, 1.9 MB [file jb.00250-21-s0001.pdf]

Supplementary Material for "Impact of artificial sputum media formulation on *Pseudomonas aeruginosa* secondary metabolite production"

Rachel L. Neve[1], Brent D. Carrillo[2] and Vanessa V. Phelan[2]

[1] Department of Immunology and Microbiology, School of Medicine, University of Colorado - Anschutz Medical Campus, Aurora, CO, 80045, USA

[2] Department of Pharmaceutical Sciences, Skaggs School of Pharmacy and Pharmaceutical Sciences, University of Colorado - Anschutz Medical Campus, Aurora, CO, 80045, USA

Table of Contents

Table S1. Citations of Commonly Used ASM Formulations 3

Table S2. Summary of compositional differences between ASM formulations 4

Figure S1. *P. aeruginosa* PAO1 growth in ASM formulations. 5

Figure S2. Feature-based molecular network of PAO1 cultures in ASM. 6

Table S3. Summary of phenotypic and chemotypic differences of PAO1 cultured in ASM formulations 7

Table S4. Metabolite annotation and mass defect from PAO1 cultures in ASM 8

Figure S3. Initial principal component analysis and its loadings plots of untargeted metabolomics data of PAO1 grown in ASM formulations. 11

Figure S4. Proportion of phenazines within the molecular family. 12

Figure S5. Structural sub-class proportion of quinolone molecular family. 12

Figure S6. Structural sub-class proportion of rhamnolipid molecular family. 13

Figure S7. PAO1 production of individual siderophore molecular families in ASM. 13

Figure S8. *P. aeruginosa* PAO1 growth in SCFM1 and PGM-SCFM1 complemented with 0.5% porcine gastric mucin (PGM-SCFM1). 14

Table S5. Metals quantified from 0.5% porcine gastric mucin (PGM) in 1X MOPS 15

Table S6. Metabolite annotation and mass defect from PAO1 cultures in SCFM1 and PGM-SCFM1 16

References 17

Table S1. Citations of commonly used ASM formulations

ASM Formulation	Citations[1]	Reference
**Soothill**	42	1
**Romling[2]**	287	2,3
**Winstanley**	91	4
**ASMDM**	103	5
**Cordwell**	35	6
**SDSU**	40	7
**SCFM1**	393	8
**SCFM2,3[3]**	180	9

[1]Citation number according to Google Scholar, accessed 5/10/2020

[2]Two associated publications: ref 2: 276 citations and ref 3: 11 citations

[3]SCFM2 and SCFM3 are described in the same publication

Table S2. Summary of compositional differences between ASM formulations

ASM Formulation (# Components)	Mucin % (w/v)	DNA % (w/v)	Iron Added (µM)	Amino Acid Source	Lipid Source	NOTES
**Soothill (7)**	0.5	0.4	-	-	Egg yolk emulsion	Lacks amino acids; includes chelator
**Romling (27)**	0.5	0.4	-	Individual; 250 mg/mL each	Egg yolk emulsion	Includes chelator
**Winstanley (26)**	0.5	0.4	-	Individual; 250 mg/mL each	Egg yolk emulsion	Includes chelator
**ASMDM (28)**	0.5	0.14	-	Individual; 250 mg/mL each	Egg yolk emulsion	Includes chelator and BSA
**Cordwell (8)**	1	0.14	Ferritin (2.85)	Casamino acids	Egg yolk emulsion	Lacks Asn, Cys, Gln, and Trp
**SDSU (8)**	2	0.14	Ferritin (2.85)	MEM amino acids	Egg yolk emulsion	Lacks Cys and Gln
**SCFM1 (32)**	-	-	$FeSO_4$ (3.6)	Individual; As measured (sputum)	DOPC	Lacks Asn and Gln; includes Orn, Mg^{2+}, and Ca^{2+}
**SCFM2 (36)**	0.5	0.06	$FeSO_4$ (3.6)	Individual; As measured (sputum)	DOPC	Lacks Asn and Gln; includes Orn, Mg^{2+}, Ca^{2+}, *N*-acetyl glucosamine
**SCFM3 (42)**	0.5	0.06	$FeSO_4$ (3.6)	Individual; As measured (sputum)	DOPC	Lacks Asn and Gln; includes Orn, Mg^{2+}, Ca^{2+}, *N*-acetyl glucosamine, *p*-aminobenzoic acid, various nucleosides

BSA: bovine serum albumin; DOPC: 1,2-Dioleoyl-sn-glycero-3-phosphocholine

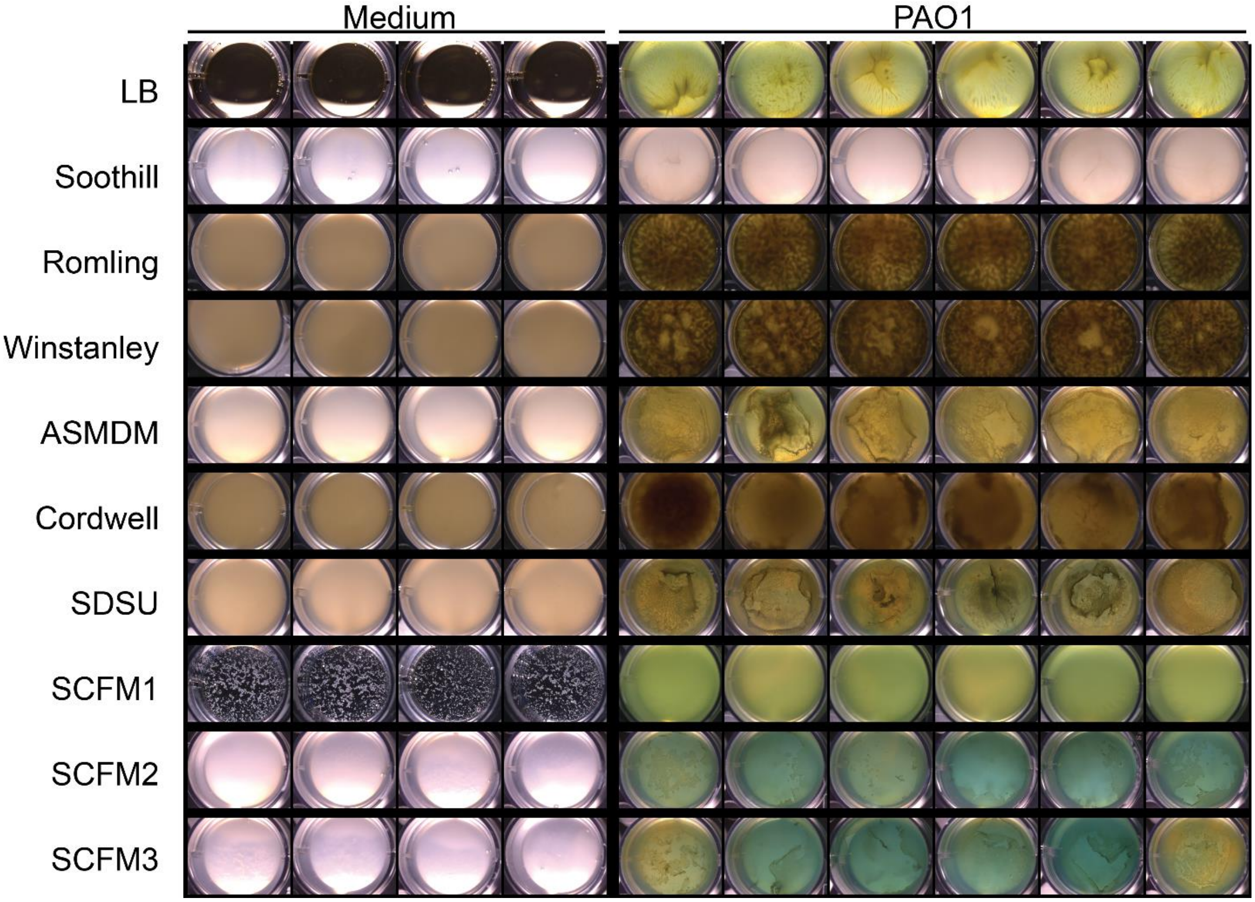

Figure S1. ***P. aeruginosa*** **PAO1 growth in ASM formulations.** Photographs of replicate control media wells and PAO1 growth in LB and ASM formulations.

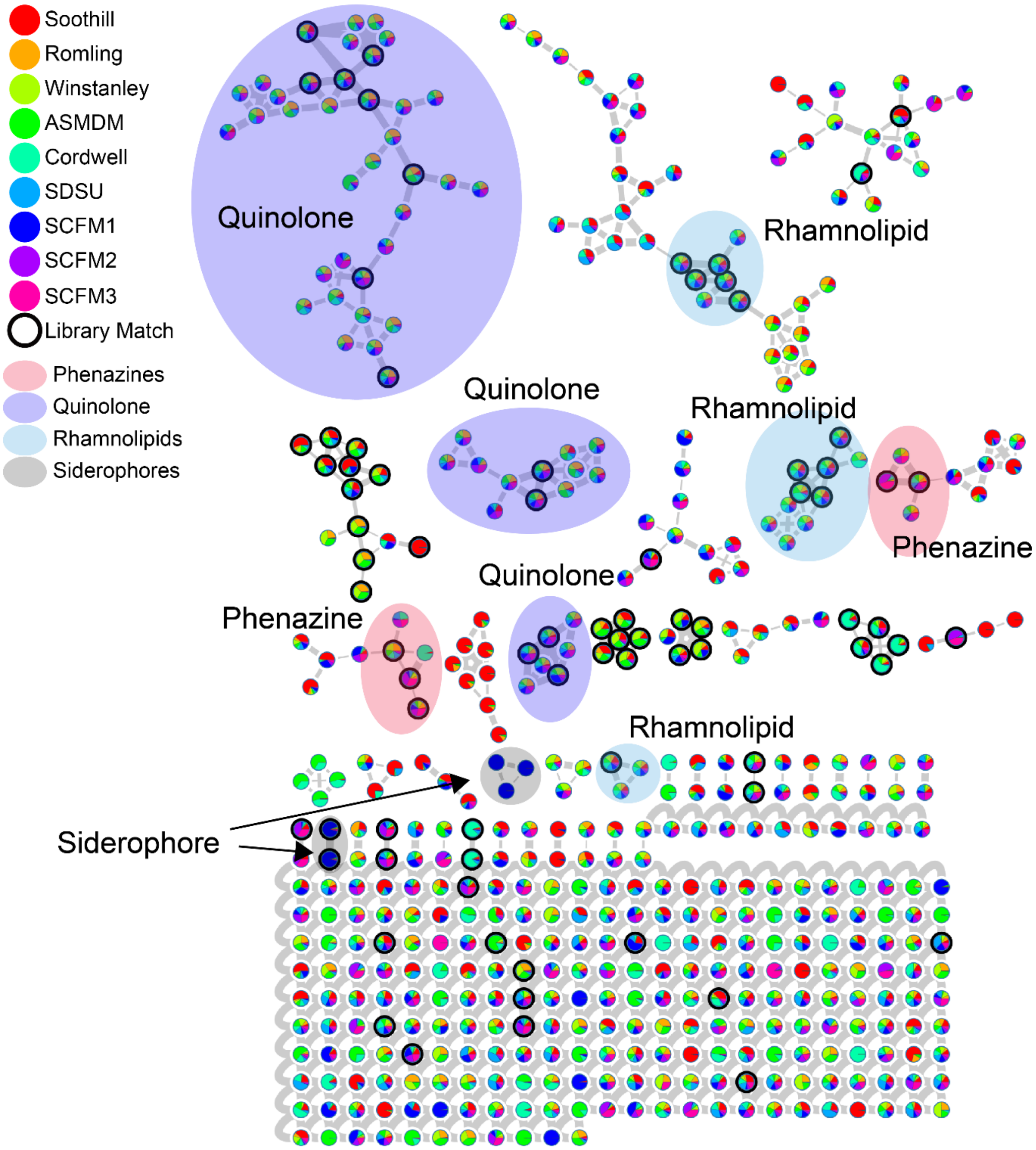

Figure S2. Feature-based molecular network of PAO1 cultures in ASM. Data includes extracts from PAO1 cultures in all ASM formulations (n=12 biological replicates per formulation). Nodes represent distinct molecular ions (precursor mass and LC retention time). The width of the edges represent the similarity of the MS/MS fragmentation of the connected nodes, while the node color represents the relative molecular ion abundance between ASM formulations. Black circles indicate a spectral match between the data and known *P. aeruginosa* metabolites represented by MS/MS spectra within the GNPS spectral libraries. Nodes corresponding to the four molecular families discussed in the main text are highlighted.

Table S3. Summary of phenotypic and chemotypic differences of PAO1 cultured in ASM formulations

ASM Formulation	Color	Phenotype	Phenazine Levels[a] (1-HP:PYO:PCA)	Quinolone Levels[a] (HHQ:HQNO:PQS)	Rhamnolipid Levels[a] (MonoRLs:DiRLs)	Siderophore Levels
**Soothill**	Translucent	Turbidity	+ (3:5:91)	+ (76:19:3)	++ (37:63)	-
**Romling**	Brown	Floating macrostructures	+++ (28:21:49)	++ (59:18:17)	++ (44:54)	-
**Winstanley**	Brown	Floating macrostructures	+++ (22:28:49)	++ (54:25:15)	++ (43:57)	-
**ASMDM**	Tan-green	Macrostructure with tunnels at air liquid interface to turbidity below	+++ (18:11:67)	++ (67:15:15)	++ (42:58)	-
**Cordwell**	Brown	Macrostructure with no distinct structures	+++ (16:21:62)	++ (49:35:8)	++ (33:67)	-
**SDSU**	Green	Macrostructure at center of well, small tendrils	+++ (16:32:52)	+ (56:33:6)	++ (30:70)	-
**SCFM1**	Yellow-green	Small clumps of growth below surface	++ (3:54:42)	++ (57:34:3)	++ (42:58)	++++
**SCFM2**	Blue	Structured at air liquid interface and biofilms on well bottom	++++ (2:57:39)	++ (45:41:4)	+ (40:60)	-
**SCFM3**	Blue	Structured at air liquid interface and biofilms on well bottom	++++ (2:58:38)	++ (49:37:5)	+ (43:57)	-

[a]Relative comparison of total production to medium with lowest levels (Soothill for phenazines, SDSU for quinolones, SCFM2 for rhamnolipids). Ratios are proportion of individual phenazines; HHQ-, HQNO-, and PQS-type quinolones; and mono- to di-RLs.

Table S4. Metabolite annotation and mass defect from PAO1 cultures in ASM

Molecular Family	ID	Annotation	Adduct	Measured *m/z*	Calculated *m/z*	Mass Defect (ppm)	Annotation Level[#]
Phenazine	10	1-HP	[M+H]	197.0702	197.0709	-3.55	1
Phenazine	16	PYO	[M+H]	211.0858	211.0866	-3.79	1
Phenazine	226	PYO	[M+H]	211.086	211.0866	-2.84	1
Phenazine	34	PCN	[M+H]	224.0813	224.0818	-2.23	1
Phenazine	8	PCA	[M+H]	225.0651	225.0659	-3.55	1
Quinolone	76	C5-HQ	[M+H]	216.1377	216.1383	-2.78	2
Quinolone	391	C6-HQ	[M+H]	230.1535	230.1539	-1.74	2
Quinolone	72	C5-QNO	[M+H]	232.1325	232.1332	-3.02	2
Quinolone	1	C7-HQ (HHQ)	[M+H]	244.1688	244.1696	-3.28	1
Quinolone	189	C6-QNO	[M+H]	246.1482	246.1489	-2.84	2
Quinolone	117	C8:1-HQ	[M+H]	256.169	256.1696	-2.34	2
Quinolone	51	C7:1-HQ-OH	[M+H]	258.1482	258.1489	-2.71	3
Quinolone	32	C8-HQ	[M+H]	258.1845	258.1852	-2.71	2
Quinolone	3	C7-QNO (HQNO)	[M+H]	260.1638	260.1645	-2.69	1
Quinolone	4	PQS	[M+H]	260.1638	260.1645	-2.69	1
Quinolone	5	C9:1-HQ	[M+H]	270.1845	270.1852	-2.59	2
Quinolone	436	C9:1-HQ	[M+H]	270.1847	270.1852	-1.85	2
Quinolone	2	C9-HQ (NHQ)	[M+H]	272.2001	272.2009	-2.94	2
Quinolone	48	C8-QNO	[M+H]	274.1795	274.1802	-2.55	2
Quinolone	399	C9:2-PQS	[M+H]	284.1633	284.1645	-4.22	3
Quinolone	105	C10:1-HQ	[M+H]	284.2003	284.2009	-2.11	2
Quinolone	369	C10:1-HQ	[M+H]	284.2005	284.2009	-1.41	2
Quinolone	179	C9:1-PQS	[M+H]	286.1795	286.1802	-2.45	3
Quinolone	11	C9:1-QNO<br>C9:1-PQS	[M+H]	286.1795	286.1802	-2.45	2
Quinolone	291	C9:1-HQ-OH	[M+H]	286.1795	286.1802	-2.45	3

Quinolone	7	C9-QNO (NQNO)	[M+H]	288.1951	288.1958	-2.43	2
Quinolone	44	C9-PQS	[M+H]	288.1951	288.1958	-2.43	2
Quinolone	77	C9-HQ-OH	[M+H]	288.1953	288.1958	-1.73	3
Quinolone	205	C11:2-HQ	[M+H]	296.1995	296.2009	-4.73	3
Quinolone	9	C11:2-HQ	[M+H]	296.2004	296.2009	-1.69	3
Quinolone	225	C11:1-HQ	[M+H]	298.2156	298.2165	-3.02	2
Quinolone	236	C11:1-HQ	[M+H]	298.2157	298.2165	-2.68	2
Quinolone	307	C10:1-QNO<br>C10:1-PQS	[M+H]	300.1955	300.1958	-1.00	2
Quinolone	19	C11-HQ	[M+H]	300.2315	300.2322	-2.33	2
Quinolone	324	C9-QNO-OH<br>C9-PQS-OH	[M+H]	304.1901	304.1907	-1.97	3
Quinolone	288	C12:1-HQ	[M+H]	312.2318	312.2322	-1.28	2
Quinolone	31	C11:1-QNO<br>C11:1-PQS	[M+H]	314.2109	314.2115	-1.91	2
Quinolone	83	C11-QNO	[M+H]	316.2265	316.2271	-1.90	2
Quinolone	388	C13:2-HQ	[M+H]	324.2317	324.2322	-1.54	3
Quinolone	17	C13:2-HQ	[M+H]	324.2317	324.2322	-1.54	3
Quinolone	63	C13:1-HQ	[M+H]	326.2473	326.2478	-1.53	2
Quinolone	172	C13-HQ	[M+H]	328.2629	328.2635	-1.83	2
Quinolone	67	C13:2-QNO<br>C13:2-PQS	[M+H]	340.2269	340.2271	-0.59	3
Quinolone	283	C13:1-QNO<br>C13:1-PQS	[M+H]	342.2422	342.2428	-1.75	3
Quinolone	160	C15:2-HQ	[M+H]	352.2627	352.2635	-2.27	3
Quinolone	315	C15:1-HQ	[M+H]	354.2780	354.2791	-3.10	3
Quinolone	106	C17:1-HQ	[M+H]	382.3088	382.3104	-4.18	3
Rhamnolipid	149	Rha-C10-C10	[M+H]	505.3371	505.3371	0	1
Rhamnolipid	30	Rha-C10-C10	[M+Na]	527.3188	527.3191	-0.57	1
Rhamnolipid	124	Rha-C10-C12:1	[M+Na]	553.3347	553.3347	0	1
Rhamnolipid	451	Rha-C10-C12	[M+H]	533.3665	533.3684	-3.56	1
Rhamnolipid	139	Rha-C10-C12	[M+Na]	555.3504	555.3504	0	1

Rhamnolipid	40	Rha-Rha-C10-C10	[M+H]	651.3948	651.3950	-0.31	1
Rhamnolipid	133	Rha-Rha-C10-C10	[2M+K+H]	670.3689	670.3730	-6.12	1
Rhamnolipid	45	Rha-Rha-C10-C10	[M+Na]	673.3767	673.3770	-0.45	1
Rhamnolipid	154	Rha-Rha-C10-C12:1	[M+H]	677.4109	677.4107	0.30	1
Rhamnolipid	110	Rha-Rha-C10-C12:1	[M+Na]	699.3928	699.3926	0.29	1
Rhamnolipid	88	Rha-Rha-C10-C12	[M+H]	679.4263	679.4263	0	1
Rhamnolipid	71	Rha-Rha-C10-C12	[M+Na]	701.408	701.4083	-0.43	1
Siderophore	300	PCH	[M+H]	325.0673	325.0675	-0.62	2
Siderophore	298	PCH	[M+H]	325.0673	325.0675	-0.62	2
Siderophore	426	PVD E	[M+2H]	667.3094	667.3102	-1.20	2
Siderophore	428	PVD E	[M+3H]	445.2085	445.2092	-1.57	2
Siderophore	410	PVD E + Fe	[M+2H]	693.7660	693.7660	0	2
Siderophore	411	PVD E + Fe	[M+3H]	462.8470	462.8464	1.30	2
Siderophore	424	Ferribactin	[M+2H]	676.3333	676.3337	-0.59	3
Siderophore	378	Ferribactin	[M+3H]	451.2249	451.2249	0	3
Quaternary amine	29	Benzethonium	[M]	412.3218	412.3210	1.94	3

[#] Annotation level according to ref. 10

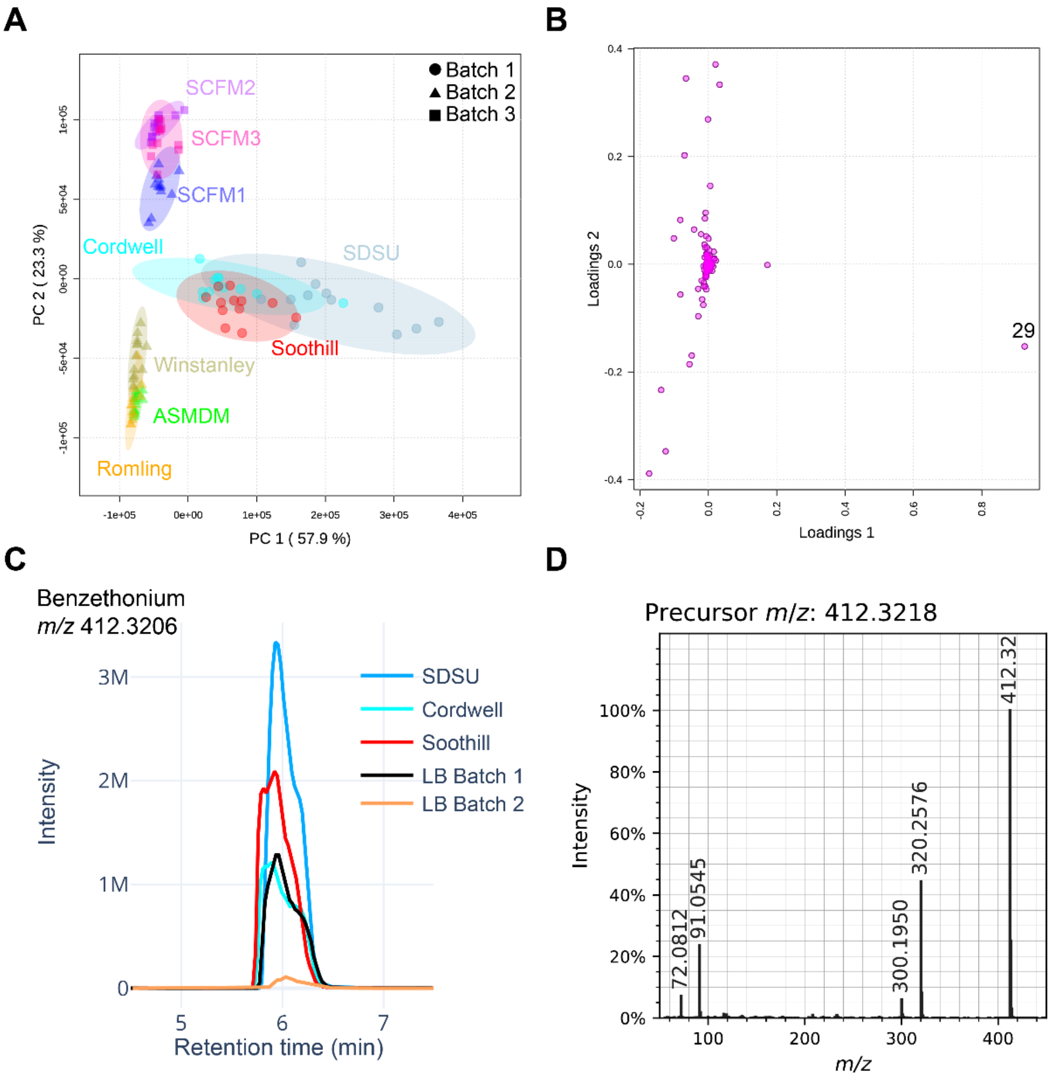

Figure S3. Initial principal component analysis and its loadings plots of untargeted metabolomics data of PAO1 grown in ASM formulations. (A) Principal component analysis scores plot of PAO1 samples, colored by medium and drawn with 95% confidence ellipses. The three processing batches are indicated by shapes. Data points representing the 12 replicate PAO1 metabolomics samples per medium were closely clustered and distinct clusters were observed between 1) Romling, Winstanley, and ASMDM ASM samples; 2) SCFM1, SCFM2 and SCFM3 samples; and Soothill, Cordwell and SDSU ASM samples. (B) Loadings plot from untargeted LC-MS/MS based principal component analysis of PAO1 in ASM formulations. Molecular ion 29 (*m/z* 412.3206) was the dominant metabolite underlying the differentiation of the ASM formulations along principle component 1 (PC1), leading to batch-based clustering of Soothill ASM with Cordwell and SDSU in the PCA. (C) Extracted ion chromatograms of m/z 412.3206 from SDSU, Cordwell, Soothill, LB Batch 1, and LB Batch 2, indicating that this metabolite was detected primarily in Batch 1 samples. Based upon exact mass, this molecular ion was putatively annotated as benzethonium. (D) The annotation of benzethonium was confirmed by comparing the MS/MS spectrum to the Metlin metabolite database (ref 11).

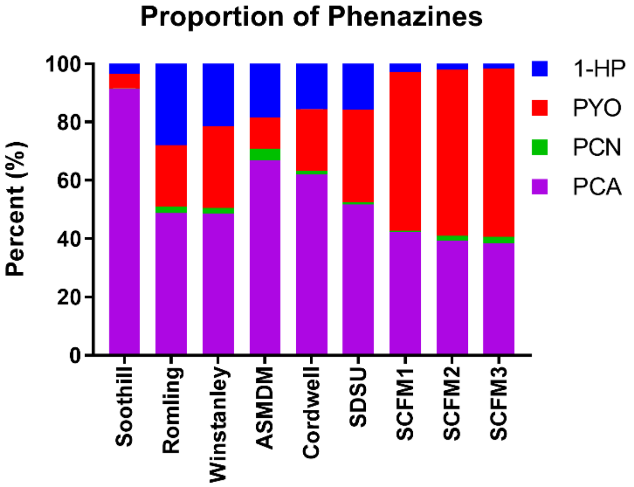

Figure S4. Proportion of phenazines within the molecular family. The normalized area under the curve for molecular ions identified as phenazines were compared to identify proportional differences in phenazine production by PAO1 grown in different ASM. 1-HP: 1-hydroxyphenazine; PYO: pyocyanin; PCN: phenazine-1-carboxamide; PCA: phenazine-1-carboxylic acid.

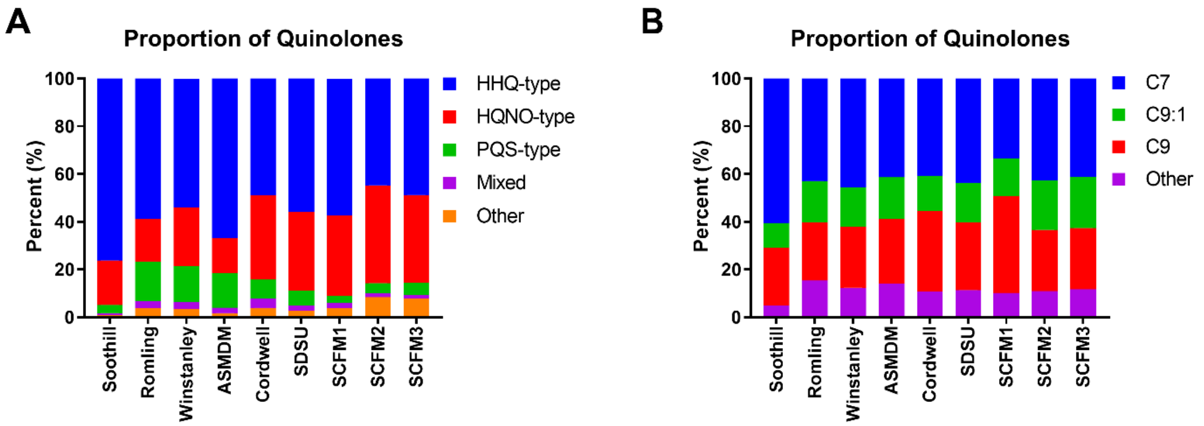

Figure S5. Structural sub-class proportion of quinolone molecular family. The normalized area under the curve for all molecular ions identified as quinolones were summed and compared proportionally to identify alterations in structural sub-class quinolone production by PAO1 grown in different ASM. (A) The percentage of total quinolone production based upon HHQ-, HQNO-, and PQS-type structural sub-class. HHQ: 2-heptyl-4-quinolone; PQS: 2-heptyl-3,4-dihydroxyquinoline, Pseudomonas quinolone signal; HQNO: 2-heptyl-4-hydroxyquinoline N-oxide. Molecular ions that could not be distinguished based upon MS/MS fragmentation patterns are referred to as 'Mixed'. Molecular ions identified as containing a hydroxyl group at an unknown position within the molecule are designated as 'Other'. (B) The percentage of total quinolones produced, comprised of different alkyl chain lengths. C7 quinolones include HHQ, HQNO,

and PQS. Quinolones with alkyl chain lengths other than C7, C9:1, and C9 are labeled as ‘Other’.

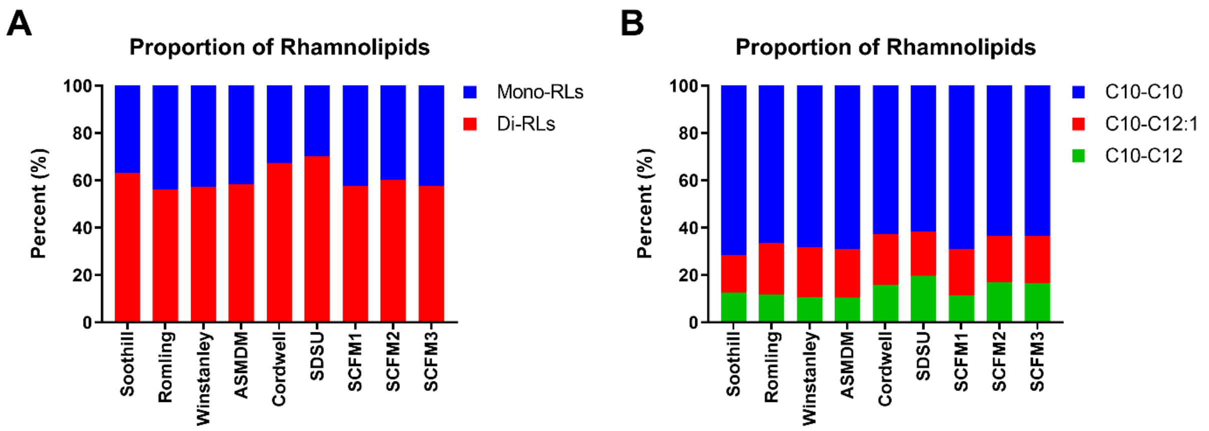

Figure S6. Structural sub-class proportion of rhamnolipid molecular family. The normalized area under the curve for all molecular ions identified as rhamnolipids were summed and compared proportionally to identify alterations in structural sub-class rhamnolipid production by PAO1 in different ASM. (A) The percentage of total rhamnolipids produced that incorporate one or two rhamnolipids are labeled as mono-RLs and di-RLs, respectively. (B) The percentage of total rhamnolipids produced, comprised of different fatty acids, including C10-C10, C10-C12:1, or C10-C12.

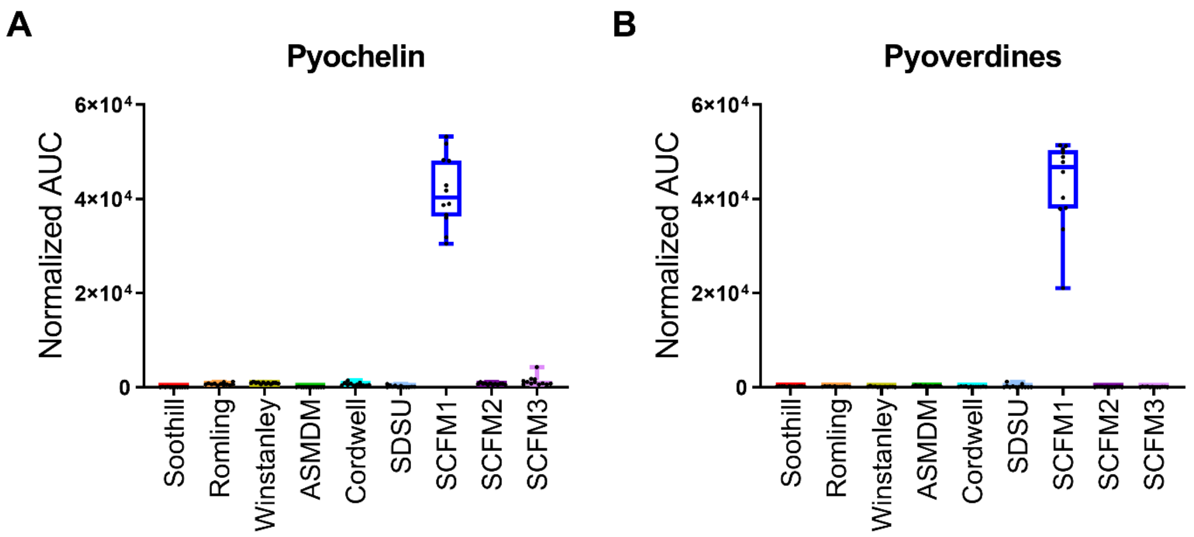

Figure S7. PAO1 production of individual siderophore molecular families in ASM. Sum of the total normalized area under the curve (AUC) for molecular ions identified as siderophores from each ASM formulation. Box plots represent the 25-75th percentile, with a line at the median. Error bars indicate the minimum to maximum. Individual samples

values shown (n=12 biological replicates per ASM formulation). (A) Pyochelin (PCH). (B) Pyoverdine (PVD) molecular family.

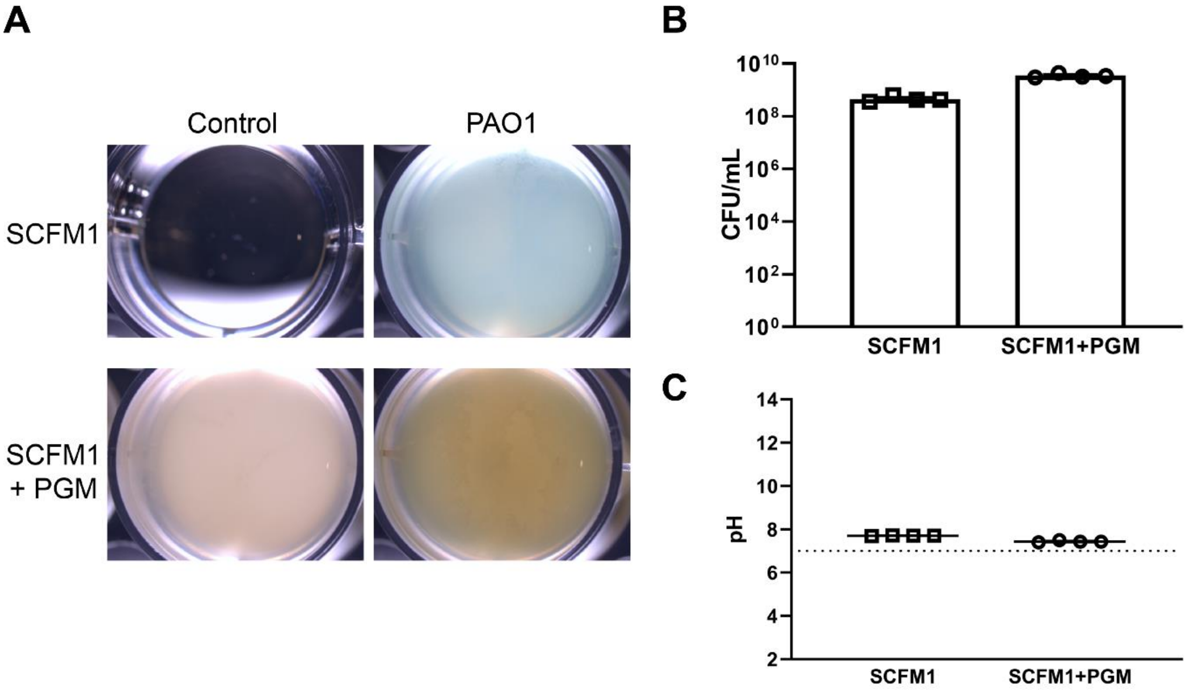

Figure S8. *P. aeruginosa* PAO1 growth in SCFM1 and PGM-SCFM1 complemented with 0.5% porcine gastric mucin (PGM-SCFM1). (A) Representative phenotypes of PAO1 grown statically in 2 mL of each medium (n = 4) in 24 well plates for 72 hours at 37 °C. (B) PAO1 growth (CFU/mL, n=4) in SCFM1 and SCFM + PGM. C) pH measurement of replicates (n=4) after growth in the media. Starting pH of all cultures was 7.0 ± 0.1.

Table S5. Metals quantified from 0.5% porcine gastric mucin (PGM) in 1X MOPS

**Metal** ppt (SD)	**1X MOPS***	**0.5% PGM***
**Na**	1.48E+08 (2.1E+06)	3.52E+08 (2.31E+07)
**Mg**	1.88E+04 (2.04E+03)	3.53E+07 (2.19E+06)
**K**	-	1.10E+07 (6.89E+05)
**Ca**	1.31E+05 (4.78E+03)	2.62E+07 (1.28E+06)
**Cr**	-	2.63E+05 (1.61E+04)
**Mn**	-	3.51E+05 (2.30E+04)
**Fe**	1.34E+05 (2.6E+04)	8.55E+06 (6.64E+05)
**Co**	-	2.61E+03 (1.75E+02)
**Ni**	-	5.04E+04 (2.89E+03)
**Cu**	-	6.96E+05 (4.12E+04)
**Zn**	-	1.62E+06 (9.32E+04)
**Se**	-	2.27E+04 (1.05E+03)
**Mo**	-	2.22E+03 (2.229E+02)

*n=6 technical replicates per sample type. All values are parts per trillion (ppt) followed by standard deviation of the technical replicates

Table S6. Metabolite annotation and mass defect from PAO1 cultures in SCFM1 and PGM-SCFM1

Molecular Family	ID	Annotation	Adduct	Measured *m/z*	Calculated *m/z*	Mass Defect (ppm)	Annotation Level[#]
Phenazine	32	1-HP	[M+H]	197.0705	197.0709	-2.03	1
Phenazine	13	PYO	[M+H]	211.0859	211.0866	-3.32	1
Phenazine	26	PCA	[M+H]	225.0653	225.0659	-2.67	1
Quinolone	1	C7-HQ (HHQ)	[M+H]	244.1691	244.1696	-2.05	1
Quinolone	3	C7-QNO (HQNO)	[M+H]	260.1641	260.1645	-1.54	1
Quinolone	124	C9:1-HQ	[M+H]	270.1846	270.1852	-2.22	2
Quinolone	7	C9:1-HQ	[M+H]	270.1847	270.1852	-1.85	2
Quinolone	2	C9-HQ (NHQ)	[M+H]	272.2004	272.2009	-1.84	2
Quinolone	504	C9:1-QNO/ C9:1-PQS	[M+H]	286.1794	286.1802	-2.8	2
Quinolone	24	C9:1-HQ-OH	[M+H]	286.1797	286.1802	-1.75	2
Quinolone	4	C9-QNO (NQNO)	[M+H]	288.1955	288.1958	-1.04	2
Rhamnolipid	313	Rha-C10-C10	[M+H]	505.3358	505.3371	-2.57	1
Rhamnolipid	90	Rha-C10-C10	[M+Na]	527.3188	527.3191	-0.57	1
Rhamnolipid	343	Rha-C10-C12	[M+Na]	555.3493	555.3504	-1.98	1
Rhamnolipid	61	Rha-Rha-C10-C10	[M+H]	651.3933	651.3950	-2.61	1
Rhamnolipid	47	Rha-Rha-C10-C10	[M+Na]	673.3759	673.3770	-1.63	1
Rhamnolipid	417	Rha-Rha-C10-C12:1	[M+H]	677.4094	677.4107	-1.92	1
Rhamnolipid	269	Rha-Rha-C10-C12:1	[M+Na]	699.3916	699.3926	-1.43	1
Rhamnolipid	176	Rha-Rha-C10-C12	[M+H]	679.4255	679.4263	-1.18	1
Rhamnolipid	102	Rha-Rha-C10-C12	[M+Na]	701.407	701.4083	-1.85	1
Siderophore	15	PCH	[M+H]	325.0669	325.0675	-1.85	2
Siderophore	8	PCH	[M+H]	325.067	325.0675	-1.54	2
Siderophore	122	PVD E	[M+2H]	667.3084	667.3102	-2.7	2
Siderophore	506	Ferribactin	[M+2H]	676.3315	676.3337	-3.25	3
Siderophore	609	Ferribactin	[M+3H]	451.2237	451.2249	-2.66	3

References

1. Ghani M, Soothill JS. 1997. Ceftazidime, gentamicin, and rifampicin, in combination, kill biofilms of mucoid Pseudomonas aeruginosa. Canadian Journal of Microbiology 43:999-1004.

2. Sriramulu DD. 2010. Artifical Sputum Medium. Protocol Exchange doi:10.1038/protex.2010.212.

3. Sriramulu DD, Lunsdorf H, Lam JS, Romling U. 2005. Microcolony formation: a novel biofilm model of Pseudomonas aeruginosa for the cystic fibrosis lung. Journal of Medical Microbiology 54:667-676.

4. Kirchner S, Fothergill JL, Wright EA, James CE, Mowat E, Winstanley C. 2012. Use of Artificial Sputum Medium to Test Antibiotic Efficacy Against Pseudomonas aeruginosa in Conditions More Relevant to the Cystic Fibrosis Lung. Jove-Journal of Visualized Experiments doi:ARTN e3857 10.3791/3857.

5. Fung C, Naughton S, Turnbull L, Tingpej P, Rose B, Arthur J, Hu HH, Harmer C, Harbour C, Hassett DJ, Whitchurch CB, Manos J. 2010. Gene expression of Pseudomonas aeruginosa in a mucin-containing synthetic growth medium mimicking cystic fibrosis lung sputum. Journal of Medical Microbiology 59:1089-1100.

6. Hare NJ, Soe CZ, Rose B, Harbour C, Codd R, Manos J, Cordwell SJ. 2012. Proteomics of Pseudomonas aeruginosa Australian Epidemic Strain 1 (AES-1) Cultured under Conditions Mimicking the Cystic Fibrosis Lung Reveals Increased Iron Acquisition via the Siderophore Pyochelin. Journal of Proteome Research 11:776-795.

7. Quinn RA, Whiteson K, Lim YW, Salamon P, Bailey B, Mienardi S, Sanchez SE, Blake D, Conrad D, Rohwer F. 2015. A Winogradsky-based culture system shows an association between microbial fermentation and cystic fibrosis exacerbation (vol 9, pg 1024, 2015). Isme Journal 9:1052-1052.

8. Palmer KL, Aye LA, Whiteley M. 2007. Nutritional cues control Pseudomonas aeruginosa multicellular Behavior in cystic fibrosis sputum. Journal of Bacteriology 189:8079-8087.

9. Turner KH, Wessel AK, Palmer GC, Murray JL, Whiteley M. 2015. Essential genome of Pseudomonas aeruginosa in cystic fibrosis sputum. Proceedings of the National Academy of Sciences of the United States of America 112:4110-4115.

10. Sumner LW, Amberg A, Barrett D, Beale MH, Beger R, Daykin CA, Fan TWM, Fiehn O, Goodacre R, Griffin JL, Hankemeier T, Hardy N, Harnly J, Higashi R, Kopka J, Lane AN, Lindon JC, Marriott P, Nicholls AW, Reily MD, Thaden JJ, Viant MR. 2007. Proposed minimum reporting standards for chemical analysis. Metabolomics 3:211-221.

11. Guijas C, Montenegro-Bruke JR, Domingo-Almenara X, Palermo A, Warth B, Hermann G, Koellensperger G, Huan T, Uritboonthai W, Aisporna AE, Wolan DW, Spilker

ME, Benton HP, Siuzdak G. 2018. METLIN: A technology platform for identifying knowns and unknowns. Analytical Chemistry 90:3156-3164.
